# Nanosensitizer-assisted sonodynamic therapy for breast cancer

**DOI:** 10.1186/s12951-025-03311-3

**Published:** 2025-04-07

**Authors:** Jing Yu, Jun-Rui Hu, Yi Tian, Yu-Meng Lei, Hai-Man Hu, Bing-Song Lei, Ge Zhang, Yao Sun, Hua-Rong Ye

**Affiliations:** 1 Department of Medical Ultrasound, China Resources & Wisco General Hospital, Wuhan University of Science and Technology, Wuhan, 430080 China; 2 Department of Pharmacy, Union Hospital, Tongji Medical College, Huazhong University of Science and Technology, Wuhan, 430022 China; 3 Department of Electrical and Electronic Engineering, Hubei University of Technology, Wuhan, 430068 China; 4 National Key Laboratory of Green Pesticides, College of Chemistry, Central China Normal University, Wuhan, 430079 China

**Keywords:** Sonodynamic therapy, Sonosensitizer, Nanomedicine, Breast cancer

## Abstract

Breast cancer is the most commonly diagnosed cancer worldwide. Despite advancements in therapeutic modalities, its prognosis remains poor owing to complex clinical, pathological, and molecular characteristics. Sonodynamic therapy (SDT) is a promising approach for tumor elimination, using sonosensitizers that preferentially accumulate in tumor tissues and are activated by low-intensity ultrasound to produce reactive oxygen species. However, the clinical translation of SDT faces challenges, including the limited efficiency of sonosensitizers and resistance posed by the tumor microenvironment. The emergence of nanomedicine offers innovative strategies to address these obstacles. This review discusses strategies for enhancing the efficacy of SDT using sonosensitizers, including rational structural modifications, improved tumor-targeted enrichment, tumor microenvironment remodeling, and imaging-guided therapy. Additionally, SDT-based multimodal therapies, such as sono-chemotherapy, sono-immunotherapy, and sono-photodynamic therapy, and their potential applications in breast cancer treatment are summarized. The underlying mechanisms of SDT in breast cancer are briefly outlined. Finally, this review highlights current challenges and prospects for the clinical translation of SDT, providing insights into future advancements that may improve therapeutic outcomes for breast cancer.

**Graphical abstract:**

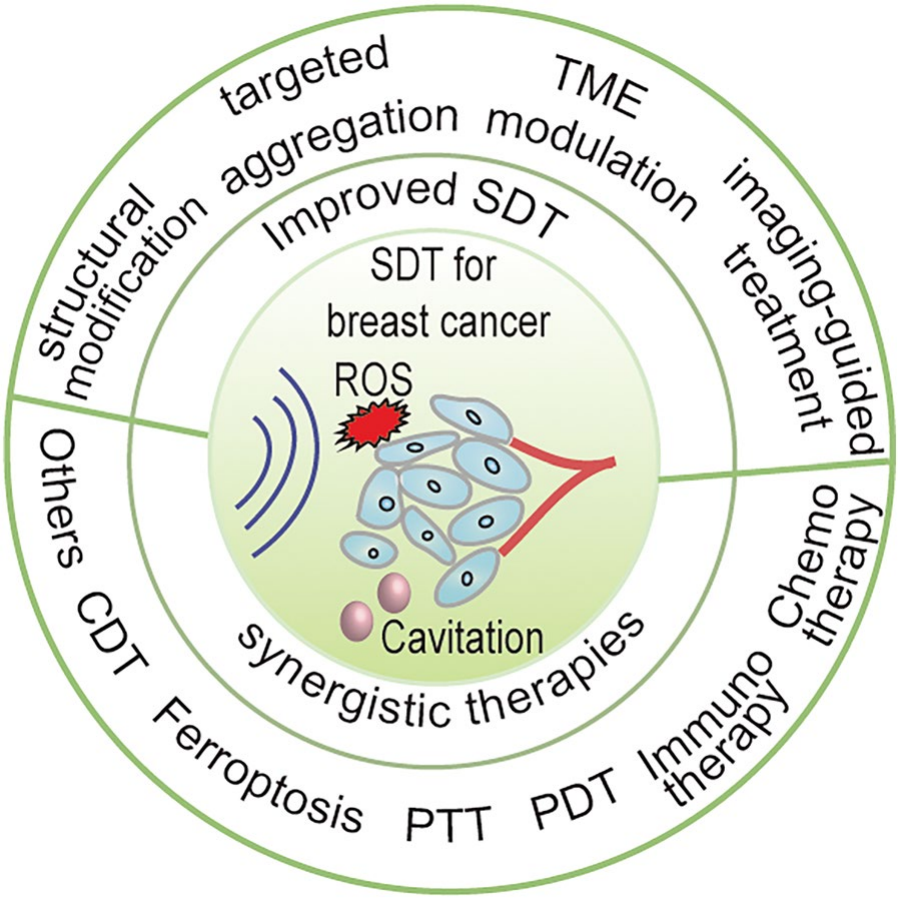

## Background

Breast cancer has become the most commonly diagnosed cancer worldwide, surpassing lung cancer [1, 2]. Moreover, it is the second leading cause of cancer-related deaths among women, particularly in transitioning countries [3, 4]. In 2020, the number of new cases of breast cancer in China reached 0.42 million, posing a severe threat to women's health [5]. Breast cancer is a heterogeneous disease with many subtypes with unique clinical, pathological, and molecular characteristics. Additionally, breast cancer treatment requires a comprehensive plan based on the pathological type, molecular classification, and disease stage. Breast cancer treatments include surgery, chemotherapy, radiotherapy, immunotherapy, endocrine therapy, and targeted therapy [6]. These modalities have improved the survival rate of patients with breast cancer. For example, the 5-year survival rate of patients with non-invasive breast cancer is 99%; however, the undesirable prognosis and severe side effects should not be ignored. When invasive breast cancer spreads to the regional lymph nodes, the 5-year survival rate is 85%. Furthermore, the survival rate decreases to 27% once distant metastasis occurs [7]. The poor prognosis is primarily attributed to the heterogeneous tumor microenvironment (TME) and resistance of cancer cell resistance to therapy [8]. Moreover, the “cold” TME inhibits immune cell activation, further reducing the effectiveness of immunotherapy-based treatments [9]. Therefore, there is an urgent need to develop novel and effective therapeutic strategies.

Sonodynamic therapy (SDT), a new strategy for tumor treatment, has non-invasive, highly controllable, deep tissue penetration, and targeted ability. SDT employs ultrasound (US) to activate sonosensitizers, which selectively accumulate in tumor tissues. Upon activation, these sonosensitizers generate reactive oxygen species (ROS), targeting and destroying tumor cells while minimizing damage to surrounding normal tissues from damage [10–13]. SDT typically uses low-intensity pulsed US, with an intensity range of 0.5 to 5 Wcm^−2^ and a frequency range of 0.035 to 3 MHz. SDT holds promise for overcoming the limitations of conventional breast cancer treatments. First introduced for tumor treatment in 1990, SDT has mainly been clinically tested for brain tumors, whereas its application for breast cancer remains in the preclinical stage [14–20]. This may be owing to the inherent limitations of current sonosensitizers for breast cancer, such as low ROS production efficiency and poor tumor targeting [21]. For example, organic sonosensitizers, such as porphyrins and their derivatives, have certain issues, including poor tumor accumulation and low sonostability [22], whereas inorganic sonosensitizers have issues with biological safety and a quick combination of electrons (e^−^) and holes (h^+^) that can greatly limit the ROS generation capability [23]. Furthermore, hypoxia and high glutathione (GSH) concentrations in the tumor microenvironment (TME) greatly limit the efficacy of SDT [24–26].

The development of nanotechnology has provided new methods to address the limitations of SDT [27]. Several new strategies have been explored to promote the efficacy of SDT in breast cancer treatment, including the use of multifunctional nanosonosensitizers or nanoplatforms. Multifunctional nanosonosensitizers are typically composed of sonosensitizers, tumor-targeting ligands, and imaging units for imaging-guided therapy. The introduction of nanoparticles provides numerous strategies for improving the accumulation and utilization rates of sonosensitizers in breast cancer, in addition to overcoming resistance to SDT owing to hypoxia and high GSH levels in the TME. Imaging agents in multifunctional nanosonosensitizers can realize the personalized delivery and monitoring of SDT for precise breast cancer treatment. In addition, combination therapy based on SDT can generate remarkable synergistic effects, which can compensate for the limitations of single-mode SDT and significantly improve the efficacy of breast cancer treatment.

Although multiple reviews have been published on the classification, fabrication, and therapeutic use of sonosensitizers [21, 23, 28–32], there has been no systematic review on nanosonosensitizers-enhanced SDT for treating breast cancer. This review systematically summarizes the mechanisms of SDT, improved SDT strategies, and joint strategies of SDT, in addition to the related clinical applications for breast cancer. In addition, the current challenges in the field and foregrounds for clinical translation are discussed. We believe this review will provide directions for the future development of SDT for breast cancer treatment.

## Mechanism of SDT

**Fig. 1:**
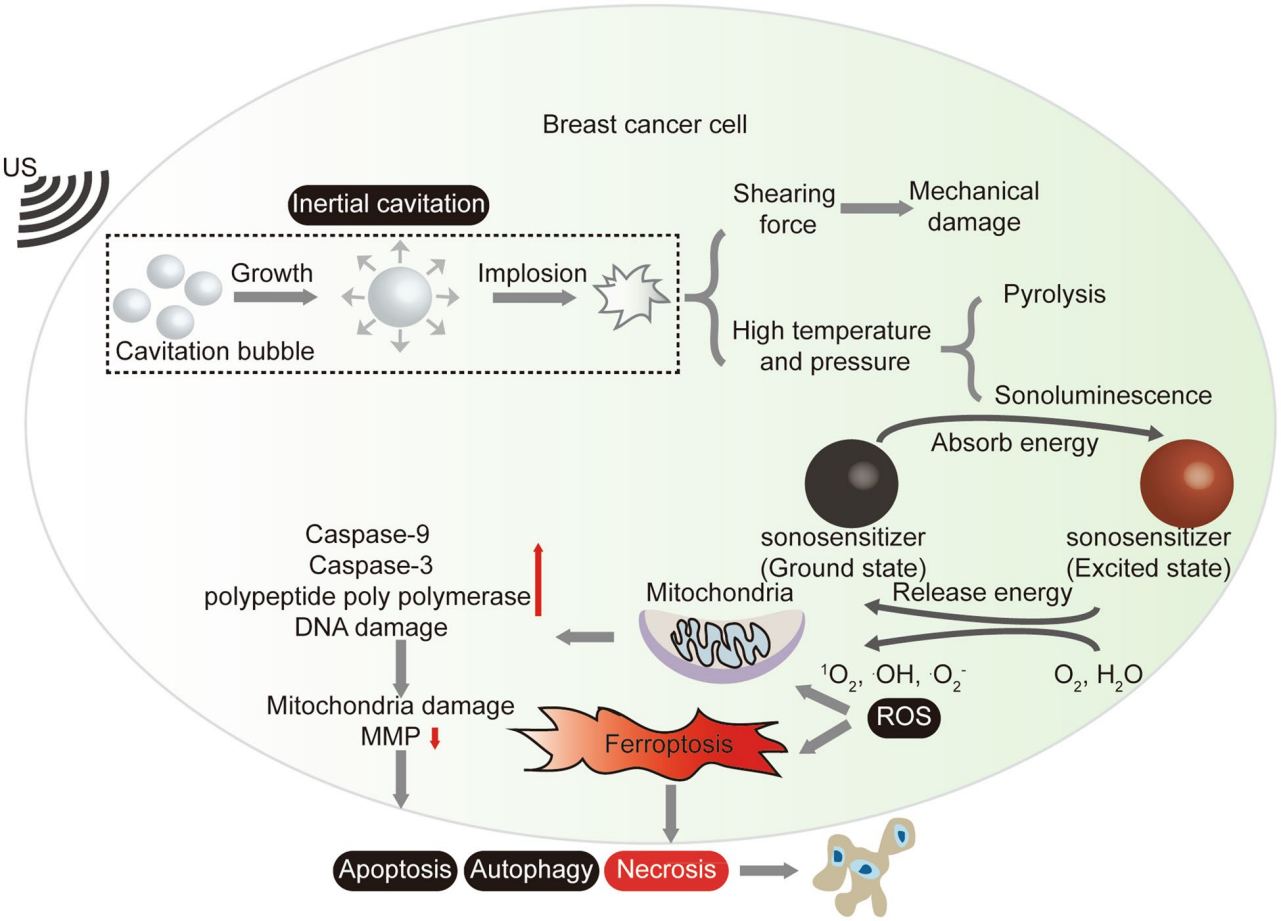
Schematic illustration of the SDT mechanisms in breast cancer

In recent years, the mechanisms of SDT have been extensively discussed [21, 33]. Nevertheless, its precise working principle remains unclear owing to the complexity of this process. Currently, the most widely accepted mechanisms reported in the literature are the cavitation effect induced by the US [29] and the production of ROS (Fig. 1) [34].

### Cavitation effect

Under US stimulation, the liquid in the tissue forms microscopic air bubbles, leading to cavitation, nucleation, meritism, and the collapse of microbubbles [12, 35]. Generally, acoustic cavitation is divided into inertial and stable cavitation. The sonosensitizers in SDT are activated by inertial cavitation. Adequate acoustic intensity can cause the rapid collapse of microbubbles, releasing highly concentrated energy into the ambient environment to produce high temperature and pressure, causing damage to the cell membrane structure and enzyme avidity, potentially leading to cell death [28]. Concurrently, the collapse of microbubbles can generate high shearing forces, thereby causing mechanical damage to the cells [36].

### ROS generation

The generation of ROS mainly involves sonoluminescence and pyrolysis [37]. The energy released during the collapse of the microbubbles promotes sonoluminescence and excites sonosensitizers [28]. When the excited sonosensitizers return to the ground state, they can release energy to oxygen (O_2_), causing the formation of ROS, such as superoxide anions (·O_2_^−^) and singlet oxygen (^1^O_2_). In addition, the released energy can also cause a pyrolysis reaction of the nearby water (H_2_O) to produce hydroxyl radicals (·OH) [21, 38]. ROS can cause cellular damage and death through oxidative damage to intracellular biomolecules, including proteins, enzymes, lipids, and DNA.

### Molecular mechanisms of SDT for breast cancer treatment

Various factors, including cell type and model, sonosensitizers, and US parameters, influence the SDT mechanism. The molecular mechanisms of SDT in breast cancer treatment under different conditions have been explored in various breast cancer cell lines. Drugs that have been confirmed to be sonosensitizers include protoporphyrin IX (PpIX) [39], 5-aminolevulinic acid (5-ALA) [40], hypocrellin B [41], chlorin e6 (Ce6) [42], and titanium dioxide (TiO_2_) [43] (Table 1). Localizing sonosensitizers to subcellular organelles is important because the lifespan and diffusion length of certain free radical products produced by sonosensitizers during SDT is relatively short [39, 44]. Many studies have revealed that they are mainly concentrated in the mitochondria, indicating that the mitochondria are the primary target of SDT-induced cytotoxicity.

**Table 1:** Summary of sonosensitizers mainly concentrated in the mitochondria for traditional SDT in breast cancer treatment

Sonosensitizers	Model	US power	References
PpIX	MDA-MB-231	1.1 MHz, 1 Wcm^−2^,1 min	[39]
5-ALA	EMT6	1 MHz, 2.15 Wcm^−2^, 2 min	[40]
hypocrellin B	MDA-MB-231	0.72 Wcm^−2^	[41]
Ce6	MDA-MB-231	1 MHz, 1 Wcm^−2^	[42]
TiO_2_	MCF-7	1 Wcm^−2^, 30 s	[43]
Pt-Cy	4T1	3 MHz, 0.3 Wcm^−2^, 20 min	[45]

Under US irradiation, ROS generated by the activation of sonosensitizers can damage the mitochondrial membrane, resulting in a decline in the mitochondrial membrane potential (MMP) and mitochondrial swelling. Simultaneously, apoptosis-related proteins, such as caspase-3 and caspase-9, and DNA damage increase, collectively inducing apoptosis of breast cancer cells. Autophagy is a double-edged sword; it can synergize, exacerbate, or antagonize apoptosis [46]. SDT can induce lysosome-dependent autophagy and provide energy for cell survival by eliminating damaged proteins and organelles, such as mitochondria, thus leading to resistance to SDT-induced cell death and promoting breast cancer progression. The therapeutic effect of SDT can be enhanced by suppressing the increase in autophagy induced by SDT [43]. For example, the application of hydroxychloroquine (HCQ), an autophagy inhibitor, significantly enhanced SDT-induced apoptosis in MCF-7 cells [43]. Furthermore, HCQ alleviated tumor hypoxia through its vascular normalization effect. Zheng et al. developed a sonoactivated liquid Z-scheme heterojunction to enhance sonodynamic mitophagy inhibition for triple-negative breast cancer (TNBC) treatment [47]. An amphipathic organic linker (PEI-PEG_5000_-C18) was used to connect the mitophagy-blocking sonosensitizer black phosphorus (BP) nanosheets with PtCu_3_ nanocages. A conjugated electron mediator (M, Cp^*^Rh(phen)Cl) positioned between the sonosensitizers facilitated electron transfer, promoting efficient ROS generation upon US stimulation. Additionally, Cu^2+^ released from PtCu_3_ accelerated BP degradation by reducing phosphorus vacancy formation energy, improving the biodegradability of BP-M-PtCu_3_ and phosphate ions production. These ions increased lysosomal pH, inhibiting the hydrolysis of damaged mitochondria within autophagic lysosomes. As a result, tumor cells were unable to sustain self-preservation under oxidative stress, leading to the effective elimination of TNBC.

Ferroptosis is a type of programmed cell death that differs from common cell death patterns, such as necrosis, apoptosis, and autophagy. Ferroptosis plays a crucial role in regulating the sensitivity of tumor cells to anti-tumor therapies [48]. Researchers have revealed that SDT-induced ROS can trigger ferroptosis and promote breast cancer cell death. Lai et al. used a platinum (II) complex coupled with a cysteine framework for SDT treatment of breast cancer. They found that characteristics related to ferroptosis occur in 4T1 breast cancer cells, such as lipid peroxidation (LPO) accumulation, decreased GSH content, and downregulation of glutathione peroxidase 4 (GPX4) [45]. In conclusion, SDT induces apoptosis in breast cancer cells mainly through ROS-induced mitochondrial damage-related changes. Autophagy and ferroptosis are also involved and can improve the sensitivity of SDT in eradicating breast cancer cells (Fig. 1).

## Improved SDT with nanosonosensitizers in breast cancer treatment

Considering the low effectiveness and accuracy of sonosensitizer accumulation at tumor sites, in addition to the complexity of the TME, a variety of strategies have been exploited to increase the efficacy of SDT for breast cancer treatment. Such strategies include modifying the structure of sonosensitizers, enhancing the tumor-targeted aggregation of sonosensitizers, and designing multifunctional sonosensitizers to overcome the TME defects of breast cancer. In addition, sonosensitizers with imaging ability are designed to accurately identify the location of tumors and realize image-guided breast cancer treatment, which not only ensures an ideal treatment window but also promotes the fusion of accurate diagnosis and treatment to achieve a secure, precise, valid, and personalized anti-cancer treatment.

### Structural modification of sonosensitizers

**Fig. 2:**
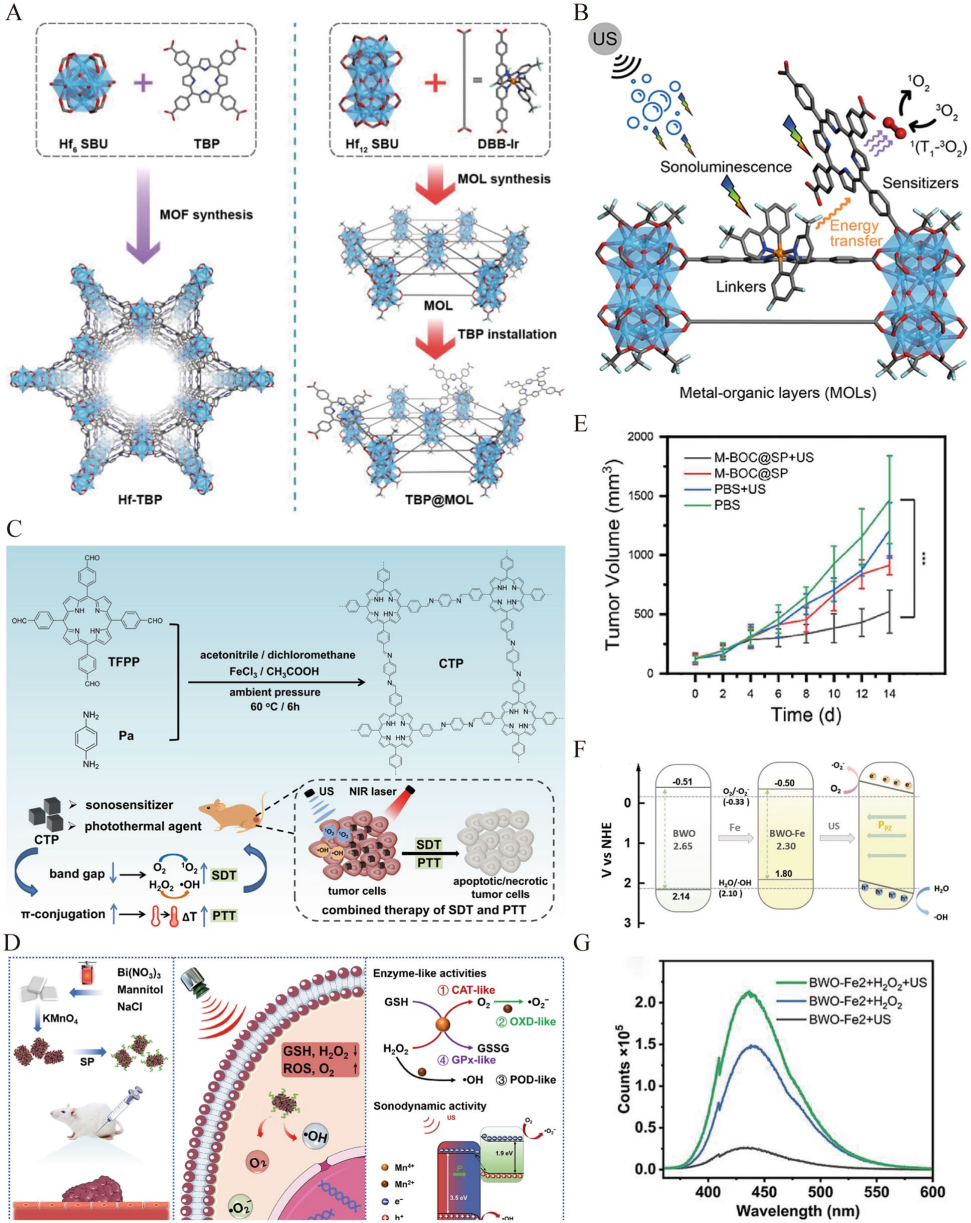
**A** The schematic diagram of the preparation of TBP@MOL for SDT. **B** The mechanism of enhanced ^1^O_2_ generation for TBP@MOL under ultrasound irradiation. Reprinted with permission from ref. [15]. Copyright 2023, Wiley. **C** Schematic illustration of the synthetic route of CTP and combined therapy. Reprinted with permission from ref. [50]. Copyright 2021, American Chemical Society. **D** Schematic illustration depicting the synthesis of M-BOC@SP NSs and mechanism of enhanced SDT against breast cancer. **E** Tumor volumes after various treatments (***P < 0.001). Reprinted with permission from ref. [16]. Copyright 2023, Elsevier. **F** Schematic illustration of energy bands of BWO-Fe NSs and band bending under ultrasound irradiation. **G** Fenton reactivity via Fe doping of BWO-Fe NSs improving sonodynamic performance.Reprinted with permission from ref. [54]. Copyright 2023, Wiley

To improve SDT efficiency, multifarious nanosystems’ structural and compositional properties have been adopted to couple with traditional sonosensitizers. Table 2 summarizes the structural modification strategies of sonosensitizers for breast cancer treatment. For example, metal–organic frameworks (MOFs) exhibit structural adjustability, porosity, and the ability to separate organic photosensitizers under high loadings [49]. Lin et al. used a two-dimensional (2D) nanoscale metal–organic layer (MOL) to couple 5,10,15,20-tetra(*p*-benzoato)porphyrin (TBP) (Fig. 2A) [15]. The newly designed sonosensitizer TBP@MOL, exhibited better bioavailability, which could overcome the aggregation-induced quenching (ACQ) of free TBP. Furthermore, they could seize broad-spectrum sonoluminescence from ultrasonic cavitation to effectively enhance the generation of singlet oxygen by promoting triplet–triplet energy transfer (TTET), achieving better sonodynamic effects on breast cancer than the three-dimensional (3D) nanoscale metal–organic framework Hf-TBP and free TBP (Fig. 2B). In addition, a cubic covalent organic framework (COF) was utilized to load the porphyrin monomer to construct CTP, which had outstanding crystallinity and unified dimensions under low temperature and ambient pressure (60 °C for 6 h). Polyvinyl pyrrolidone was used to control the cuboidal or cubic shape of the CTP and upgrade its dispersibility. Under weakly acidic conditions or after a certain period of US irradiation, the CTP exhibited good stability [50]. CTP turned out to be an excellent sonosensitizer with quantized ROS generation as the band gap was reduced for enhanced conjugation compared with porphyrin monomers. Furthermore, photothermal conversion was achieved owing to the enhancement of the π-π interaction and extension of conjugate structures (Fig. 2C). This porphyrin-based COF effectively damaged breast cancer cells with the binding of enhanced SDT and photothermal therapy (PTT).

**Table 2:** Summary of the structural modification strategies of nanosonosensitizers for breast cancer treatment

Nanosonosensitizers	Design principle of sonosensitizers	Model	US power	References
TBP@MOL	Overcome the AIQ and seize sonoluminescence from cavitation	4T1	3.4 MHz, 2 Wcm^−2^, 10 min	[15]
CTP	Improve biocompatibility and narrow band gap	4T1	1 MHz, 1.5 Wcm^−2^	[50]
M-BOC@SP NS	Improve the e^−^-h^+^ separation	4T1	1 MHz, 1.5 Wcm^−2^, 3 min	[16]
D-ZnO_x_:Gd	Inhibit e^−^-h^+^ recombination via oxygen-deficient	4T1	1 MHz, 1 Wcm^−2^	[22]
BWO-Fe NSs	Prevent e^−^-h^+^ recombination and generate ⋅OH via Fenton reactivity	4T1	1 MHz, 1.5 Wcm^−2^, 3 min	[54]

Nanomaterials can also conquer the rapid recombination of e^−^ and h^+^ in activated inorganic sonosensitizers. For instance, piezoelectric semiconductor materials can promote charge carrier separation through US-induced piezoelectric fields [51]. A new sonosensitizer, M-BOC@SP NSs, was constructed using piezoelectric bismuth oxychloride nanosheets (BiOCl NSs) modified with MnO_x_ and functionalized with soybean phospholipids (SP) to improve the biocompatibility [16]. When subjected to US irradiation, the piezoelectric field generated in the BiOCl NSs promotes the disjunction and conveyance of charge carriers, enhancing the generation of ROS. Additionally, MnO_x_ exerted multiple enzyme-like activities to downregulate intracellular glutathione levels and decompose hydrogen peroxide (H_2_O_2_) in the internal environment to produce O_2_ and ·OH, significantly promoting ROS production and reversing tumor hypoxia (Fig. 2D). M-BOC@SP NSs showed excellent biocompatibility and tumor inhibition effects in 4T1-bearing breast cancer mice under US irradiation (1 MHz, 1.5 Wcm^−2^, 3 min), providing a feasible way to improve the efficacy of SDT by using a piezoelectric nanoplatform with multiple enzyme-like activities (Fig. 2E). Integrating sonosensitizers with nanomaterials that possess good conductivity can also suppress the rapid recombination of e^−^ and h^+^; however, this structural modification may scale up the strives of synthesis and prevent long-term stability. Defect engineering can provide electron traps to suppress e^−^–h^+^ recombination [52]. Metallic doping is an effective method for introducing defects [53]. Ding et al. synthesized an iron-doped anoxic bismuth tungstate nanosheets named BWO-Fe NSs for SDT treatment, which could exert excellent inhibitory effects on refractory breast cancer (1 MHz, 1.5 Wcm^−2^, 3 min) [54]. Doping with Fe can cause oxygen defects and significantly narrow the bandgap of BWO-Fe, making it easier to activate by the US. In addition, the dynamically updated piezoelectric potential further promotes the reverse migration of e^−^ and h^+^, leading to energy band bending and promoting ROS generation (Fig. 2F). Fe doping could also bestow on BWO-Fe with Fenton reactivity, converting H_2_O_2_ into ·OH in the TME to amplify cell damage and enhance SDT (Fig. 2G).

In summary, modifying the structure of sonosensitizers with different nanomaterials can improve the efficiency of SDT, mainly by increasing the stability, cavitation effects, and ROS yield of the sonosensitizers.

### Targeted aggregation of sonosensitizers in breast cancer

To induce the targeted killing effects of tumor cells, sonosensitizers that can target aggregation and release at tumor sites have been designed through tumor-targeted ligand modification and responsive release into the TME, such as weak acidity and high GSH concentrations. Representative examples of sonosensitizers for breast cancer treatment are summarized in Table 3, which can provide optimal preparation strategies for multifunctional nanoplatforms as required.

**Table 3:** Summary of the targeted strategies of nanosonosensitizers for breast cancer treatment

Nanosonosensitizers	Design principle of sonosensitizers	Model	US power	References
Rose bengal	Target tumor by designing peptido-nanomicelles which can specifically target the integrin αvβ_3_ receptors overexpressed on tumor cellsto load sonosensitizer	MDA MB-231	1.0 MHz,duty cycle: 50%, 1.0 W cm^−2^, 5 min	[57]
Bismuth-gallic acid	Target tumor via a GSH-activated, metal-polyphenol MOF nano-prodrug	4T1	1.0 W cm^−1^, 4 min	[58]
1-NP	Target tumor and activateby redox	4T1	1 MHz, 2 Wcm^−2^, 5 min	[19]
LIP3	Target aggregation, release and activate drugsby hypoxia	4T1	1.5 Wcm^−2^, 30 s	[59]
ZDC@M NP	Improvetargeted delivery via biomimetic cell membrane and release responsive to pH	4T1	1 MHz, 1.5 Wcm^−2^, 3 min	[55]
DH-HM@BSA	Target release drugs via H-MnO_2_ carrier which can rapidly degraded in the weakly acidic TME	4T1	1 MHz, 1.5 Wcm^−2^, 3 min	[60]
Tma-BTO NPs	Trigger the self-assembly of Tma-BTO NPs in the acidic TME, generating more ROS	4T1	1 MHz, 1 Wcm^−2^, 2 min	[56]
TiO_2_@CaP NP	Degrade TiO_2_@CaP to TiO_2_ and Ca^2+^ in acidic TME, resulting in ROS generation and mitochondrial dysfunction	4T1	3 MHz, 2.1 W, 20 min	[20]

**Fig. 3:**
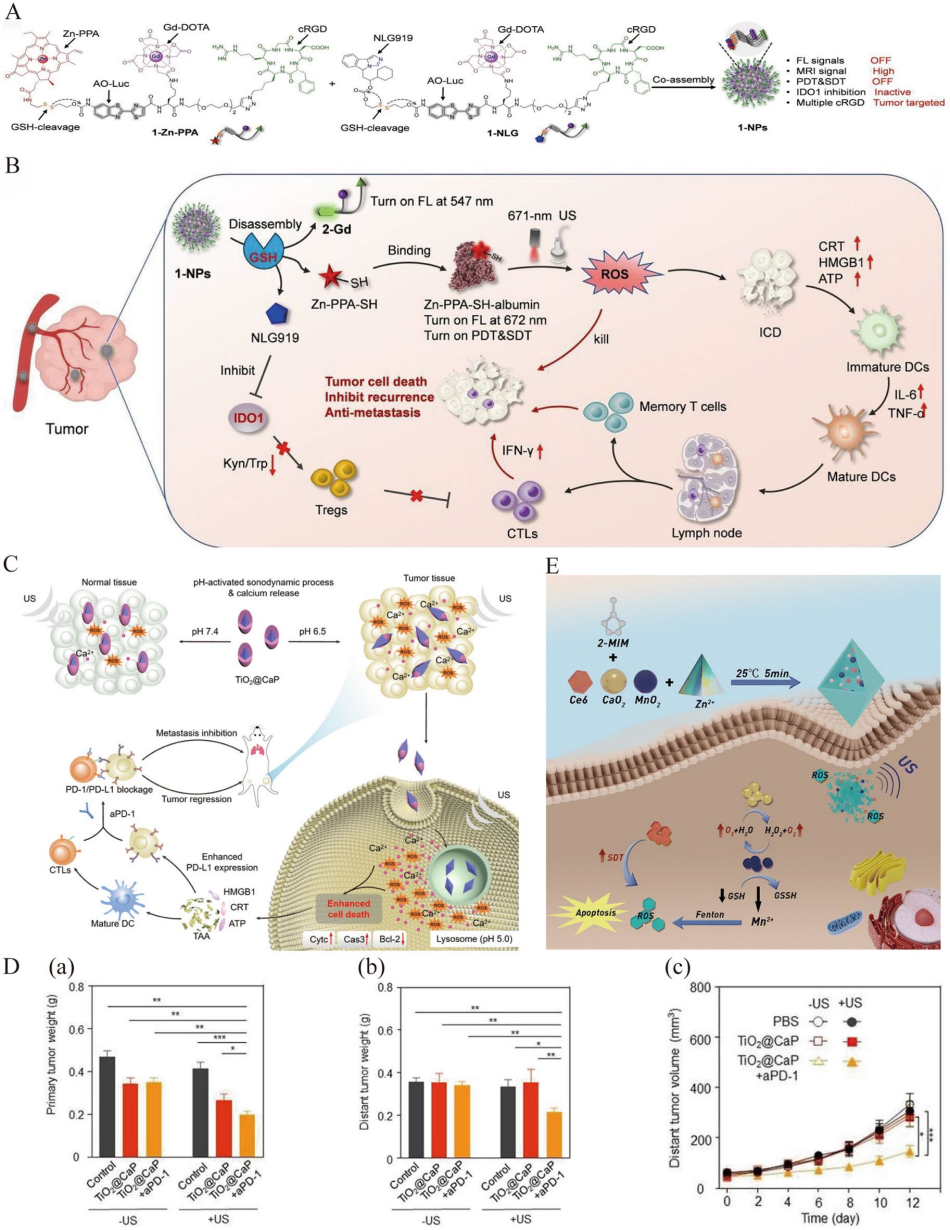
**A** Schematic illustration of the assembly of 1-NPs. **B** The mechanism of GSH-activatable sono-photodynamic immunotherapy for tumors by 1-NPs. Reprinted with permission from ref. [19]. Copyright 2023, Wiley. **C** Schematic diagram of the transformable TiO2@CaP under physiologically neutral as well as pathologically acidic condition and the mechanism for improved sono-immune therapeutic efficacy in tumor. **D** (a) Tumor weight of primary breast cancer and (b) tumor weight as well as (c) growth curves of metastatic lung cancer in 4T1 tumor-bearing mice with different treatments. Reprinted with permission from ref. [20]. Statistically significant difference: *P < 0.05; **P < 0.01; ***P < 0.001. Copyright 2021, Wiley. **E** The mechanism of efficient O2 production of MnO2/CaO2/Ce6@ZIF-8 for SDT. Reprinted with permission from ref. [68]. Copyright 2022, Royal Society of Chemistry

Liu et al. designed small-molecule probes, 1-Zn-chelated pheophorbide a (1-Zn-PPA) and 1-NLG, which were co-assembled into spherical nanoparticles to afford tumor-targeted and redox-activated nanosensitizers 1-NPs (Fig. 3A) [19]. The common components of 1-Zn-PPA and 1-NLG include the tumor-targeting ligand cyclic arginine–glycine–aspartate (cRGD) and the disulfide bond, which is sensitive to GSH. 1-NPs actively target tumors through cRGD. The high expression of GSH in tumor cells led to disulfide reduction, triggering the decomposition of the 1-NPs and discharge of the micromolecules Zn-PPA-SH and NLG919. The binding of Zn-PPA-SH with endogenous albumin generates ROS, exerting strong SDT and photodynamic therapy (PDT) effects under US (1 MHz, 2 Wcm^−2^, 5 min) and 671 nm laser irradiation. Further, immunogenic cell death (ICD) was also induced to enhance the anti-tumor immune response. NLG919 inhibits the translation of tryptophan to kynurenine by inhibiting indoleamine 2,3-dioxygenase 1 (IDO1) activity, promoting tumor infiltration of cytotoxic T lymphocytes (CTLs), thus activating anti-tumor immunity, and inhibiting tumor proliferation and metastasis. Redox-activated 1-NPs via GSH achieved combination therapy with sono-photodynamic immunotherapy for primary breast cancer (Fig. 3B). Furthermore, Ce6 was loaded into zeolitic imidazole frameworks-8 (ZIF-8), which was responsive to acidic pH [55]. This newly designed complex could not only overcome the hydrophobic defects of Ce6 but also achieve its targeted release in the acidic TME, enhancing the therapeutic effects of SDT on breast cancer (1 MHz, 1.5 Wcm^−2^, 3 min). Tan et al. developed an acidic TME-activated sonosensitizer TiO_2_@CaP NP by coating CaP with acidic degradation ability onto sonosensitizer TiO_2_ nanoparticles [20]. In an acidic TME, TiO_2_@CaP can be degraded to TiO_2_ and Ca^2+^, resulting in ROS generation and mitochondrial dysfunction, respectively. These cascade reactions promote tumor cell apoptosis and enhance ICD, thereby activating anti-tumor immunity (Fig. 3C). TiO_2_@CaP-mediated SDT combined with immune checkpoint blockade therapy induced a systemic anti-tumor immune response, which not only inhibited the treatment of primary breast cancer but also prevented distant metastasis, such as lung metastasis (Fig. 3D). Xiang et al. developed barium titanate nanoparticles (tma-BTO NPs) that self-assemble automatically in the acidic TME. In the neutral pH of normal tissues, Tma-BTO NPs remained manodisperse. However, in the acidic TME, they underwent self-assembly and accumulated, producing more ROS under US irradiation than their monodisperse counterparts, effectively inhibiting tumor progression [56].

This approach paves the way for precise SDT by designing multifunctional sonosensitizers modified with tumor-targeting ligands or responsive to the TME for controlled release or assembly, thereby minimizing side effects.

### TME modulation of breast cancer

Hypoxia and high GSH concentrations in the TME make tumors resistant to SDT therapy [61–63]. Multifunctional nanosonosensitizers which can reverse the inhibitory TME have been developed for breast cancer treatment as shown in Table 4.

**Table 4:** Summary of nanosonosensitizers with ability of TME modulation for SDT in breast cancer

Nanosonosensitizers	Design principle of sonosensitizers	Model	US power	References
SMAH	Deplete GSH to enhance SDT via self-assembled ordered metalloporphyrin nanoparticles	4T1	1 MHz, 1.0 Wcm^−2^, 2 min	[70]
IR@CPGel	Reduce GSH level through co-loading G6PD inhibitors RRx-001	MDA-MB-231	1 MHz, 1.0 Wcm^−2^, 5 min	
BTO@MNP	Produce oxygen by catalyzing the overexpressed H_2_O_2_ in TME and deplete GSH	4T1	1 MHz, 1.0 Wcm^−2^, 5 min	[71]
Chl-MOF	Generate O_2_ through Chl-mediated photosynthesis	4T1	1 MHz, 1.0 Wcm^−2^, 2 min	[72]
PPID NP	Delivery O_2_ via perfluorotributylamine	4T1	2 Wcm^−2^, 5 min	[67]
MnO_2_/CaO_2_/Ce6@ZIF-8	React with substances such as H_2_O and H_2_O_2_ in the TME to cyclically produce O_2_ by carrying MnO_2_ and CaO_2_	4T1	1 MHz, 1 Wcm^−2^, 3 min	[68]
MI-PEOz-lip	Reduce O_2_ consumption via metformin to inhibit the mitochondrial respiratory chain	MDA-MB-231	2.5 Wcm^−2^, 5 min	[69]
PE-XF/IR820@L-B NPs	Reduce O_2_ consumption by interfering with the biosynthesis of mitochondrial complex IV, thereby causing a reduction in the production of the mitochondrial ATP	4T1	1 MHz, 1 Wcm^−2^, 5 min	[73]

High levels of GSH in the TME can clear excessive ROS in tumor cells, weakening the efficiency of ROS-dependent SDT. The inhibition of glucose-6-phosphate dehydrogenase (G6PD) activity, a key enzyme in the pentose phosphate pathway, can reduce GSH levels [64]. Huang et al. prepared a self-assembled hydrogel IR@CPGelco-loaded with the sonosensitizer indocyanine green (ICG) and the G6PD inhibitor RRx-001 to inhibit breast tumor growth using SDT (1 MHz, 1 Wcm^−2^, 5 min) [65]. RRx-001 amplified ROS-mediated oxidative stress and promoted apoptosis of MDA-MB-231 breast cancer cells. In addition, hydrogels for drug delivery have good prospects for clinical transformation owing to their good biocompatibility, biodegradability, and low toxicity. Several self-assembled hydrogels have been approved for preclinical and clinical trials.

Through the production of ROS, SDT further exacerbates the hypoxic state of the TME. Improving O_2_ content in the tumor region is crucial for enhancing the efficacy of SDT. Various methods have been adopted to mitigate hypoxia in the TME, including the use of hyperbaric oxygen and nanomaterials that can transport or manufacture O_2_ [66]. However, drawbacks, such as lung damage or low O_2_ delivery efficiency, limit their clinical application. Several studies have explored more efficient and biosafe O_2_ transporters. Perfluorocarbons (PFCs) are inert synthetic materials with powerful O_2_ solubility that are extremely stable and not easily oxidized under processing, usage, and storage conditions. PFCs can dissolve large amounts of O_2_ instead of reversibly combining with O_2_. In addition, as the proportion of inspired O_2_ increases, the PFC can absorb extra O_2_ in the circulation and quickly diffuse it into tissues through the O_2_ gradient. Huang et al. developed core–shell nanoparticles with a shell composed of polylactic-*co*-glycolic acid (PLGA) loaded with IR780 and a core composed of perfluorotributylamine (PFTBA), a type of PFC, and doxorubicin (DOX) [67]. Under US irradiation (2 Wcm^−2^, 5 min), they enhanced the tumor O_2_ supply and greatly promoted SDT combined with chemotherapy to inhibit the growth of breast cancer. Manufacturing nanoparticles that can react with substances in the TME to produce O_2_ is an alternative method for alleviating tumor hypoxia. Gao et al. constructed a cascaded nanoplatform, MnO_2_/CaO_2_/Ce6@ZIF-8, which cyclically produced O_2_ by carrying MnO_2_ and CaO_2_ nanoparticles that could react with substances such as H_2_O and H_2_O_2_ in the TME [68]. ZIF-8 responds to the weakly acidic pH of the TME and releases the loaded drugs. The mechanism of the cyclic production of O_2_ for SDT was as follows (Fig. 3E): the reaction between CaO_2_ and H_2_O produced O_2_ and H_2_O_2_, whereas MnO_2_ could utilize H_2_O_2_ in the TME or produced by the above reaction to yield O_2_; then, the decomposition product H_2_O could continue to undergo the aforementioned cascade reaction with CaO_2_. In addition, reducing O_2_ consumption can overcome the hypoxia-induced resistance to tumor treatment. Metformin can inhibit mitochondrial respiration and reduce O_2_ consumption by inhibiting the avidity of mitochondrial complex I. Accordingly, Zhang et al. designed a system to encapsulate IR780 and metformin in pH-responsive phospholipids to produce liposomes (MI-PEOz-lip) [69]. Owing to the enhanced penetration and retention effect, MI-PEOz-lips aggregated specifically at tumor sites. In response to the acidic TME, metformin in liposomes inhibits the mitochondrial respiratory chain of tumor cells. IR780 released to the tumor area can produce large amounts of ROS to inhibit breast tumors under US irradiation (2.5 Wcm^−2^, 5 min). Overall, designing multifunctional sonosensitizers that can overcome the high GSH concentrations and hypoxic defects of the TME is of great significance for improving the sensitivity of breast cancer cells to SDT.

### Imaging-guided breast cancer treatment

**Fig. 4:**
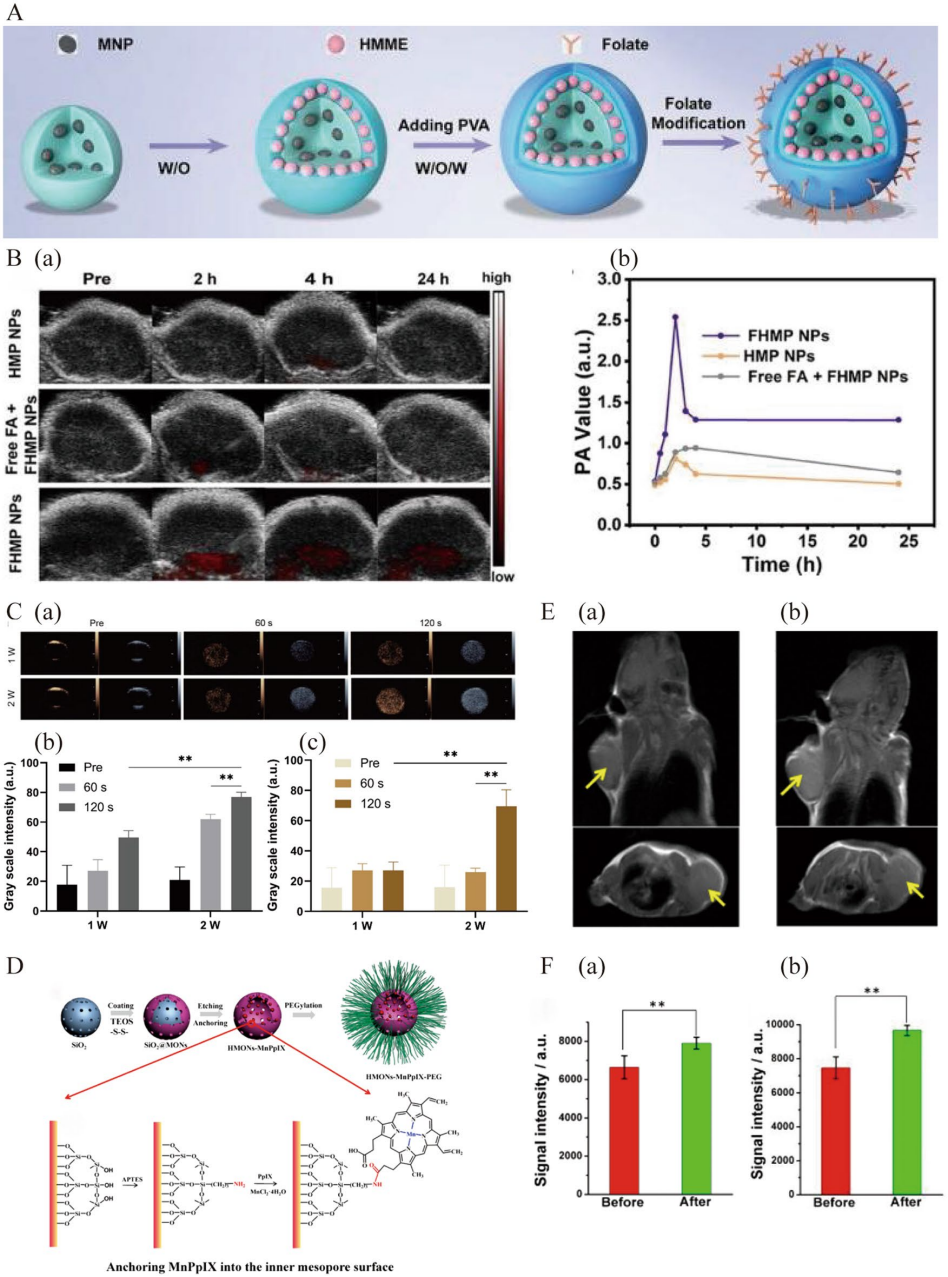
**A** Schematic illustration for the design of FHMP NPs. **B** (a) PA images and (b) PA signal at tumor regions of MDA-MB-231 tumor-bearing mice with different treatment at varied time intervals. Reprinted with permission from ref. [74]. Copyright 2018, Ivyspring International Publisher. **C** A multifunctional Ce6/PFP/DTX/PLGA nanoparticle named CPDPNP with ultrasound imaging capabilities: (a) 2D and CEUS images under different low-intensity focused ultrasound intensities and duration times. The corresponding grayscale intensity of (b) B-mode and (c) CEUS imaging at different intensities and time (**P < 0.01). Reprinted with permission from ref. [75]. Copyright 2021, Springer. **D** Schematic illustration of the synthesis of HMONs-MnPpIX-PEG. **E** The axial and coronal T1-weighted MR imaging of 4T1 tumor-bearing mice (a) before and (b) after intravenous administration of HMONs-MnPpIX-PEG. **F** (a) axial and (b) coronal T1-weighted MRI signal intensity of tumor before and after the injection of HMONs-MnPpIX-PEG (**P < 0.01). Reprinted with permission from ref. [76]. Copyright 2017, American Chemical Society

To realize accurate, effective, safe, and individualized anti-cancer treatments, multifunctional nanosonosensitizers with imaging and therapeutics have been designed for imaging-guided SDT (Table 5). Melanin nanoparticles (MNPs) exhibit a wide range of optical absorption and are ideal contrast agents for photoacoustic imaging (PA). Huang et al. constructed multifunctional FA-HMME-MNPs-PLGA (FHMP, FA: folate, HMME: hematoporphyrin monomethyl ether) nanoparticles by using biodegradable and biocompatible PLGA as a carrier to load MNPs, HMME, and tumor-targeting ligand FA (Fig. 4A) [74]. Approximately 2 h after injection, the PA signal in the tumor region reached its maximum value and gradually decreased with increasing injection time, (Fig. 4B), indicating the potential of nanosensitizers for monitoring and guiding breast cancer treatment. Zhang et al. encapsulated Ce6, perfluoropentane (PFP), and docetaxel (DTX) in PLGA to construct a novel multifunctional Ce6/PFP/DTX/PLGA nanoparticle, called CPDPNP [75]. PFP can transition from liquid to gas, which promotes the aggregation of CPDP at tumor sites and improves the efficiency of US imaging. When the US intensity reached 2 Wcm^−2^ and lasted 2 min, US imaging in B-mode and contrast-enhanced ultrasound (CEUS) was the most significant (Fig. 4C). Under the same irradiation conditions, CPDP inhibited breast tumor metastasis. Huang et al. covalently anchored PpIX to mesoporous organosilica nanoparticles (MONs). To endow them with imaging ability, paramagnetic transition metal Mn ions were chelated into porphyrin rings by relying on metalloporphyrin chemistry to construct HMON-MnPpIX-PEG (PEG: Polyethylene glycol) as a T1-weighted magnetic resonance imaging (MRI) imaging agent to guide and monitor SDT against breast cancer (Fig. 4D) [76]. Following the intravenous injection of HMON-MnPpIX-PEG into 4T1-bearing breast cancer mice, the T1-weighted positive contrast was significantly elevated in the tumors (Fig. 4E, F). In the future, for patients with different subtypes of breast cancer, nanocarriers can be modified according to specified needs to encapsulate sonosensitizers and various imaging agents. It is possible to explore an ideal treatment window for diseases and achieve an image-guided personalized treatment mode.

**Table 5:** Summary of nanosonosensitizers with imaging and therapeutics for imaging-guided SDT in breast cancer

Nanosonosensitizers	Design principle of sonosensitizers	Model	US power	References
FA-HMME-MNPs-PLGA NP	Guide SDT by photoacoustic imaging	MDA-MB-231	1 MHz, 3 Wcm^−2^, 5 min	[74]
CPDP NP	Release sonosensitizers and improve ultrasound imaging via US-responsive phase change	4T1	2 Wcm^−2^, 2 min	[75]
HMONs-MnPpIX-PEG	Facilitate the high loading of PpIX and realize MR imaging	4T1	1 MHz, 2.3 Wcm^−2^, 5 min	[76]
PGd@tNBs	Combine both MRI imaging and CEUS capabilities by incorporating gadolinium	SK-BR3		[77]
SPNH	Realize single photon emission computed tomography (SPECT) imaging via labelling with iodine-131 (^131^I)	4T1	1 MHz, 1 Wcm^−2^, 5 min	[78]

## SDT-based combination treatment of breast cancer

**Fig. 5:**
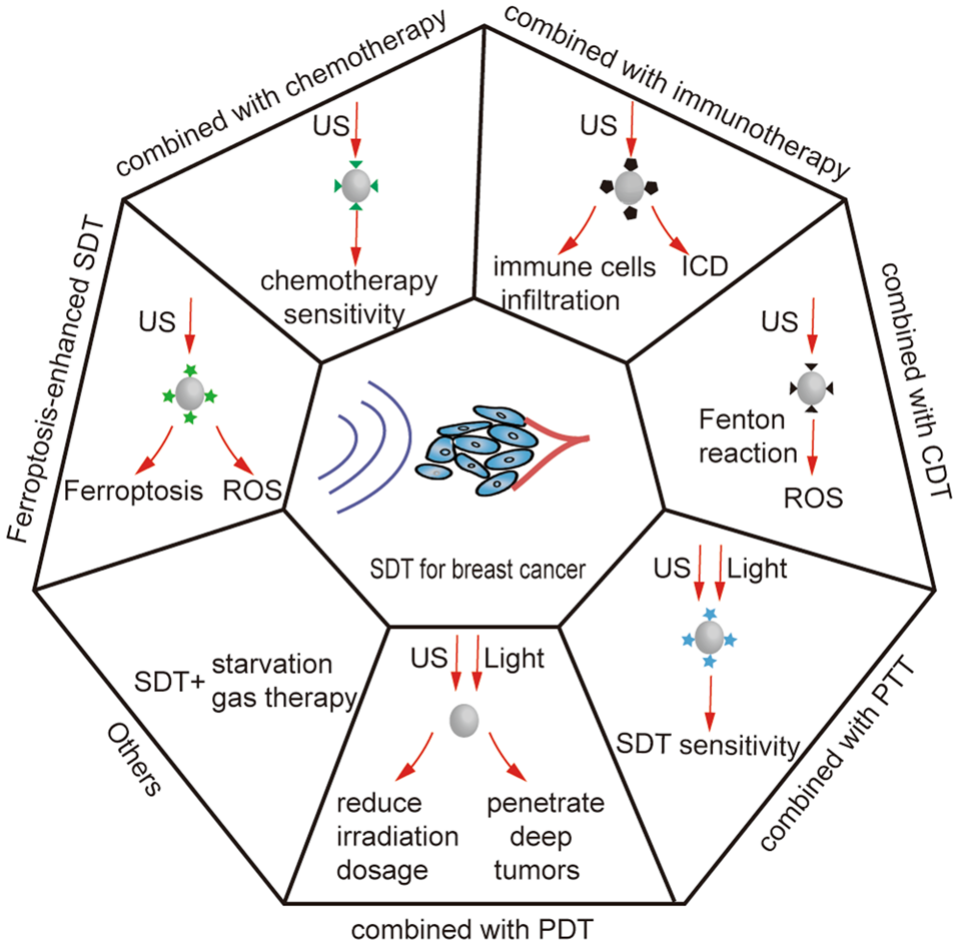
Summative scheme of combination therapy based on SDT for the treatment of breast cancer

Owing to the complexity and heterogeneity of the TME, single-modal SDT therapy often fails to achieve ideal therapeutic effects for breast cancer. Therefore, multimodal synergistic therapy has aroused widespread research interest, which can exert better anti-tumor effects with fewer drugs and the US than monotherapy. Many combination therapies, such as SDT-chemotherapy and SDT-immunotherapy, have been widely explored (Fig. 5, Table 3).

### SDT combined with chemotherapy

Chemotherapy is the first-line treatment for breast cancer without hormone receptors [79]; however, the efficacy of chemotherapy is limited owing to drug resistance and serious side effects [7]. Co-loading of chemotherapeutic drugs and sonosensitizers into nanocarriers provides a powerful platform for coordinating the synergistic interplay between SDT and chemotherapy in breast cancer treatment. The multifunctional nanoparticles can optimize the biocompatibilities and targeting of chemotherapeutic drugs, enhancing the sensitivity of tumor cells to chemotherapy and reducing side effects [80].

**Fig. 6:**
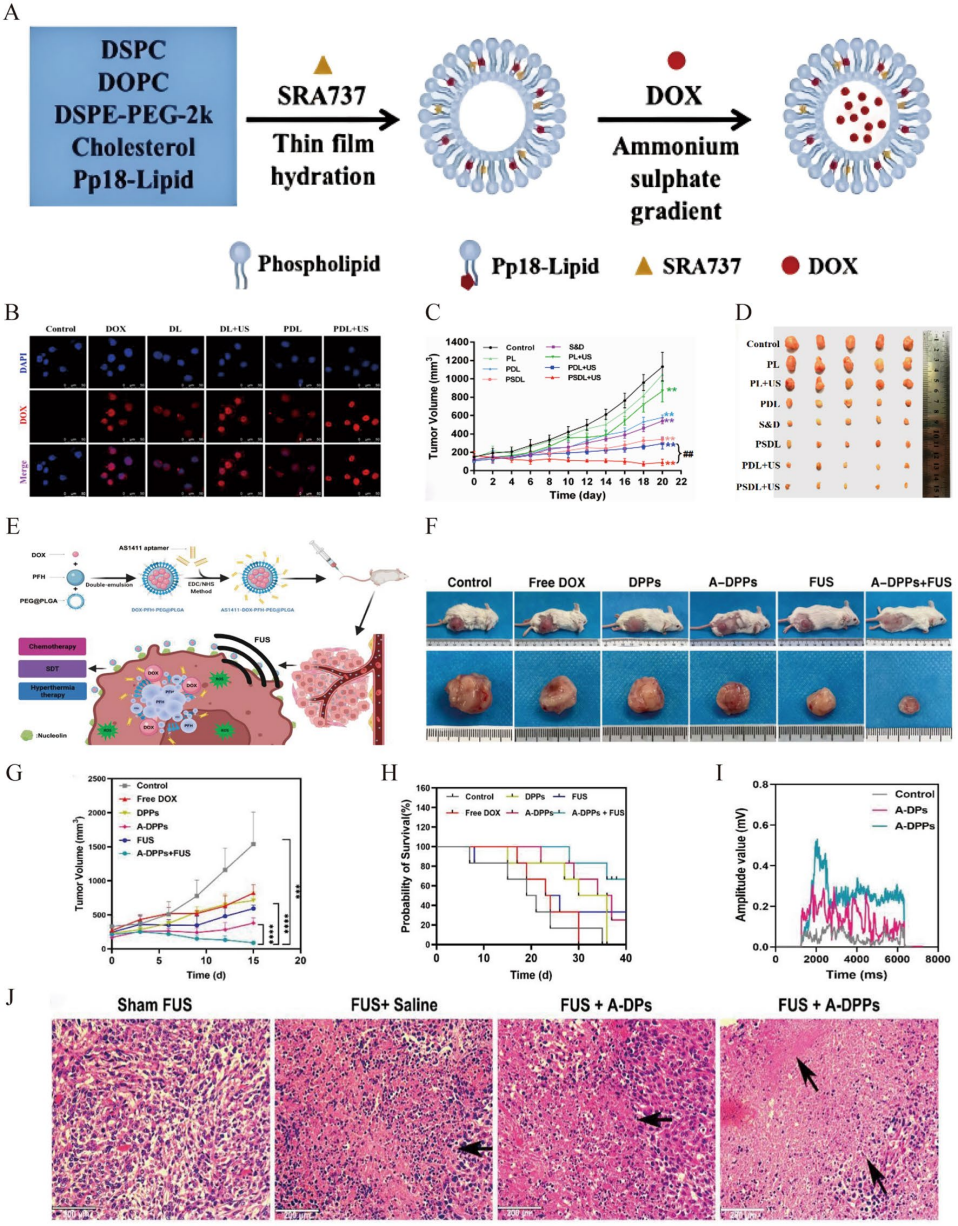
**A** Synthesis of PSDL. **B** Cell uptake of nanoparticles using the red fluorescence emission of DOX by laser confocal microscope. **C** Tumor growth curves and **D** tumor photos of MDA-MB-231 tumor-bearing nude mice after different treatments. Reprinted with permission from ref. [17]. ^**^P < 0.01, compared with control; ^##^*P* < 0.01, compared among groups. Copyright 2022, Multidisciplinary Digital Publishing Institute (MDPI). **E** Schematic demonstration of the synthesis of A-DPPs and chemotherapy and SDT combined therapy strategy. **F** The photos of 4T1-bearing mice and isolated tumors at day 15 post different treatments. **G** Tumor volumes and **H** survival curve of tumor-bearing mice after different therapies. ***P < 0.001, ****P < 0.0001. **I** The passive cavitation signals in tumor-bearing mice with different treatments. **J** Hematoxylin and eosin staining images of tumor sites to observe the coagulative necrosis areas in tumor-bearing mice with different treatments. Reprinted with permission from ref. [18]. Copyright 2022, Dove Press

To overcome the inefficient accumulation of therapeutic drugs at the tumor sites, Feng et al. co-loaded the chemotherapeutic drugs SRA737 and DOX into a liposome-like nanoporphyrin (Pp18-lipos) to construct Pp18-lipos@SRA737&DOX (PSDL) for the controlled formation of ROS (Fig. 6A). Further, US stimulation increased the release of DOX by approximately 40–70% because the sonosensitive porphyrin in Pp18-lipos can induce liposome rupture (Fig. 6B), therefore boosting chemotherapeutic effectiveness and enhancing SDT efficacy. The tumor volume inhibition rate in MDA-MB-231 tumor-bearing mice reached 92.21% in the PSDL and US group compared with SRA737/DOX alone (53.59%) (Fig. 6C, D) [17]. Imaging and targeting agents have also been introduced to achieve the precise release of agents at tumor sites and to implement imaging-guided combination therapy. For example, Kang et al. constructed AS1411-DOX/PFH-PEG@PLGA (A-DPPs) by encapsulating perfluorohexane (PFH) and DOX in the core of PEG@PLGA, the surface of which was modified with an AS1411 aptamer, a targeting ligand for tracing 4T1 breast cancer cells (Fig. 6E) [18]. US irradiation triggers the targeted release of DOX. However, PFH underwent a gas–liquid phase transition for imaging guidance of chemotherapy and SDT combined therapy, inhibiting tumor growth and prolonging the survival of TNBC mice (Fig. 6F–H). A possible mechanism may be that the enhanced inertial cavitation by A-DPPs can enhance the heating, chemotherapeutic potency, and biological effects of US at the tumor site (Fig. 6I–J) [81].

In summary, SDT combined with chemotherapy plays a crucial role in breast cancer ablation by increasing the chemotherapeutic sensitivity and SDT-induced cell apoptosis. The use of actively targeted nanomedicines provides a new strategy for the targeted delivery and release of chemotherapeutic drugs and sonosensitizers to tumor cells, thereby reducing side effects and achieving efficient tumor ablation.

### SDT combined with immunotherapy

Immunotherapy is a type of treatment in which the restrained immune system is reactivated to exert anti-tumor effects [82]. Numerous immune-based treatment methods have been explored for breast cancer, including immune checkpoint blockade (ICB) therapy, tumor vaccine immunotherapy, and adoptive T-cell immunotherapy [83, 84]. However, clinical translation still faces obstacles owing to limitations, such as low response rates and immune-related adverse events. For example, a study using pembrolizumab monotherapy for TNBC reported a response rate of only 5.3% [85]. These findings may be related to the immunosuppressive TME of breast cancer [86, 87]. SDT can induce ICD and trigger anti-tumor immunity [88–91]; however, the immune response induced by SDT was insufficient to trigger a strong immune response. Utilizing nanocarriers to combine immunotherapeutic drugs and sonosensitizers can achieve accurate drug release, improve the immune system and reduce systemic side effects, achieving SDT combined with immunotherapy for anti-tumor treatment [92, 93].

**Fig. 7:**
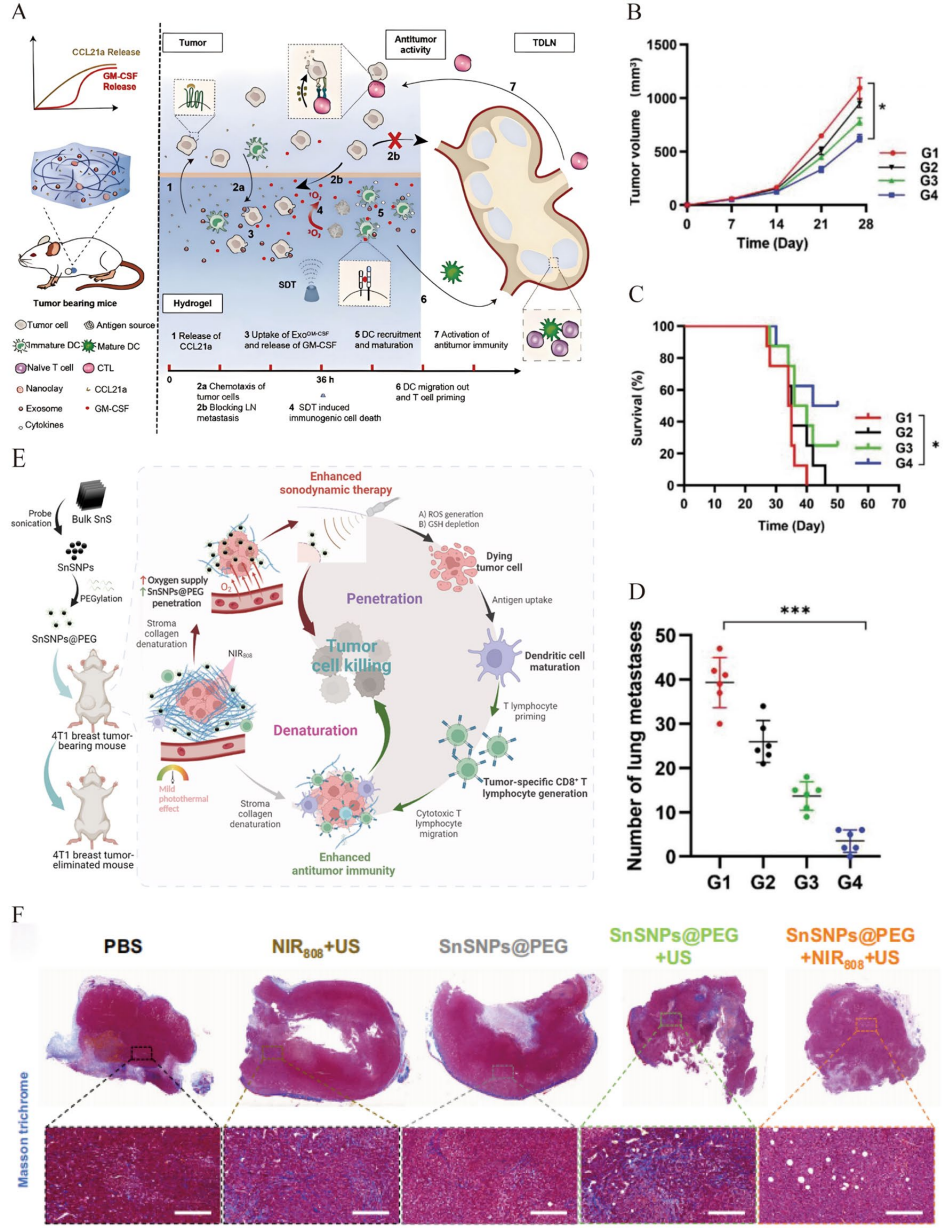
**A** Schematic illustration of the anti-tumor mechanism of CCL21a/Exo^GM−CSF+Ce6^@nanoGel. **B** Tumor growth (*P < 0.05 by two-way ANOVA) and **C** survival curves of 4T1 breast cancer mice treated with indicated hydrogels (*P < 0.01 by log-rank test). **D** The number of lung metastatic nodules at day 28 post different treatments (^***^P < 0.001 by one-way ANOVA). G1: None@nanoGel/US group; G2: CCL21a/Exo^Ctrl^@nanoGel/US group; G3: CCL21a/Exo^Ctrl+Ce6^@nanoGel/US group; G4: CCL21a/Exo^GM−CSF+Ce6^@nanoGel/US group. Reprinted with permission from ref. [94]. Copyright 2023, Wiley. **E** Schematic illustration of the synthesis of SnSNPs and their denaturation-and-penetration strategy for improved SDT. **F** The detection of content of collagen fibers via Masson’s trichrome staining of tumor tissue sections after various treatments. Reprinted with permission from ref. [97]. Copyright 2023, Nature Publishing Group

To overcome the deficiencies of tumor neoantigens and insufficient infiltration of immune cells, Ji et al. designed a modular hydrogel vaccine to trigger a strong and sustained immune response [94]. Exosomes derived from 4T1 cells contain granulocyte–macrophage colony-stimulating factor (GM-CSF) mRNA and Ce6 to form Exo^GM−CSF+Ce6^. CCL21a and Exo^GM−CSF+Ce6^ were mixed with nanoclay and gelatin methacryloyl to form theengineered hydrogel CCL21a/Exo^GM−CSF+Ce6^@nanoGel (Fig. 7A). CCL21a recruits 4T1 cells to hydrogels. The captured tumor cells engulfed the exosomes and induced Ce6-mediated SDT to produce tumor neoantigens. GM-CSF mRNA synthesizes GM-CSF in tumor cells to recruit dendritic cells (DCs) along with CCL21a. The number of mature DCs and CTLs also increased. Levels of the pro-inflammatory cytokines, including interleukin-2 (IL-2), interferon-γ (IFN-γ), and tumor necrosis factor-α (TNF-α), were significantly increased in tumor tissues, which facilitated the anti-tumor immune response. In contrast, the levels of the immune-suppressive factors, such as IL-10 and transforming growth factor-β (TGF-β), were significantly decreased. The engineered modular hydrogel vaccine promotes anti-tumor immunity through the synergistic effects of ICD induced by SDT and immune cell recruitment to efficiently inhibit the growth and lung metastasis of tumors (Fig. 7B–D).

One of the characteristics of solid tumors, such as TNBC, is the high density of the tumor extracellular matrix (ECM) [95], which limits the delivery of drugs to the tumor parenchyma and the infiltration of CTLs into the tumor [96]. Therefore, ECM degradation is a promising method for improving cancer SDT and immunotherapy. Li et al. fabricated tin monosulfide nanoparticles (SnSNPs), which have a narrow bandgap to promote the efficient separation of e^−^ and h^+^ (Fig. 7E). SnSNPs can absorb near-infrared (NIR) radiation and generate photothermal effects, improving the tumor O_2_ supply and deforming collagen in the tumor ECM to promote tumor infiltration of SnSNPs (Fig. 7F) [97]. This improves the SDT effect and enhances the infiltration of CTLs to generate anti-tumor immunity, promoting the complete eradication of TNBC tumors in situ without recurrence.

Uncontrolled tissue accumulation and the intrinsic biological activity of immunotherapy agents pose significant challenges, particularly immune-related adverse events. Among these, immune checkpoint inhibitor-associated pneumonia is the primary adverse reaction in TNBC and can lead to irreversible pulmonary fibrosis. Therefore, enhancing the spatiotemporal precision of immunotherapeutic agent release holds substantial research value for improving treatment efficacy while minimizing immune-related side effects. Li et al. utilized dendritic mesoporous silica nanoparticles (DMSNs) to encapsulate the sonosensitizer PpIX [98]. A ROS-sensitive linker wasused to covalently attach the programmed death-ligand 1 (PD-L1) inhibitor (aPD-L1) to the DMSNs. Upon US stimulation, PpIX generated ROS, which cleaved the linker, selectively releasing aPD-L1 at tumor sites. This process simultaneously triggered SDT and immunotherapy. This sono-immnotherapy strategy demonstrated remarkable efficacy in tumor ablation, immune response activation, and pulmonary fibrosis prevention.

SDT combined with immunotherapy has been explored clinically. Gc protein-derived macrophage-activating factor (GcMAF), which activates macrophages, can be used in cancer immunotherapy [99]. After treatment with GcMAF (intramuscular injection of 0.5 mL, twice a week, 21 times in total), SDT (intravenous injection of 25 mg Ce6 and oral administration of 10 mg/kg 5-ALA, 19 times in total), and exemestane (25 mg/day), the right axillary tumor, pleural effusion, and pleural nodule of a 55-year-old patient with advanced breast cancer completely disappeared [100]. The percentage and number of monocytes increased significantly, whereas the number of tumor markers decreased rapidly. This indicates that this combination therapy is a non-invasive and well-tolerated treatment.

In conclusion, nanomaterials, nanomaterial-based drug delivery systems offer promising strategies for overcoming the immunosuppressive TME, providing new hope for effectively inducing anti-tumor immunity and achieving synergistic therapeutic effects. However, the design and fabrication of these systems often involve complex processes and high costs, limiting their widespread application. Therefore, developing a simple yet effective sono-immunotherapy reagent and strategy has become a key research focus in this field.

### SDT combined with PDT

PDT can produce ROS that induce tumor cell death by stimulating photosensitizers with specific wavelengths of therapeutic light [101–103]. PDT has been approved by the United States Food and Drug Administration (FDA) for the treatment of diverse cancers and premalignant conditions [104]. However, owing to the finite penetration depth of light, deep tumors exhibit a poor response to PDT alone. SDT can penetrate deep-seated malignant tumors and compensate for the limitations of PDT. In addition, numerous sensitizers such as IR780, curcumin, and indocyanine green can not only generate PDT effect under light irradiation, but also SDT effect under US irradiation. Therefore, the combination of sonodynamic and photodynamic therapy (SPDT) can produce more notable anti-cancer effects than any single treatment and can reduce the energy of US or light and the dosage of sensitizers through this dual-use approach of single material.

**Fig. 8:**
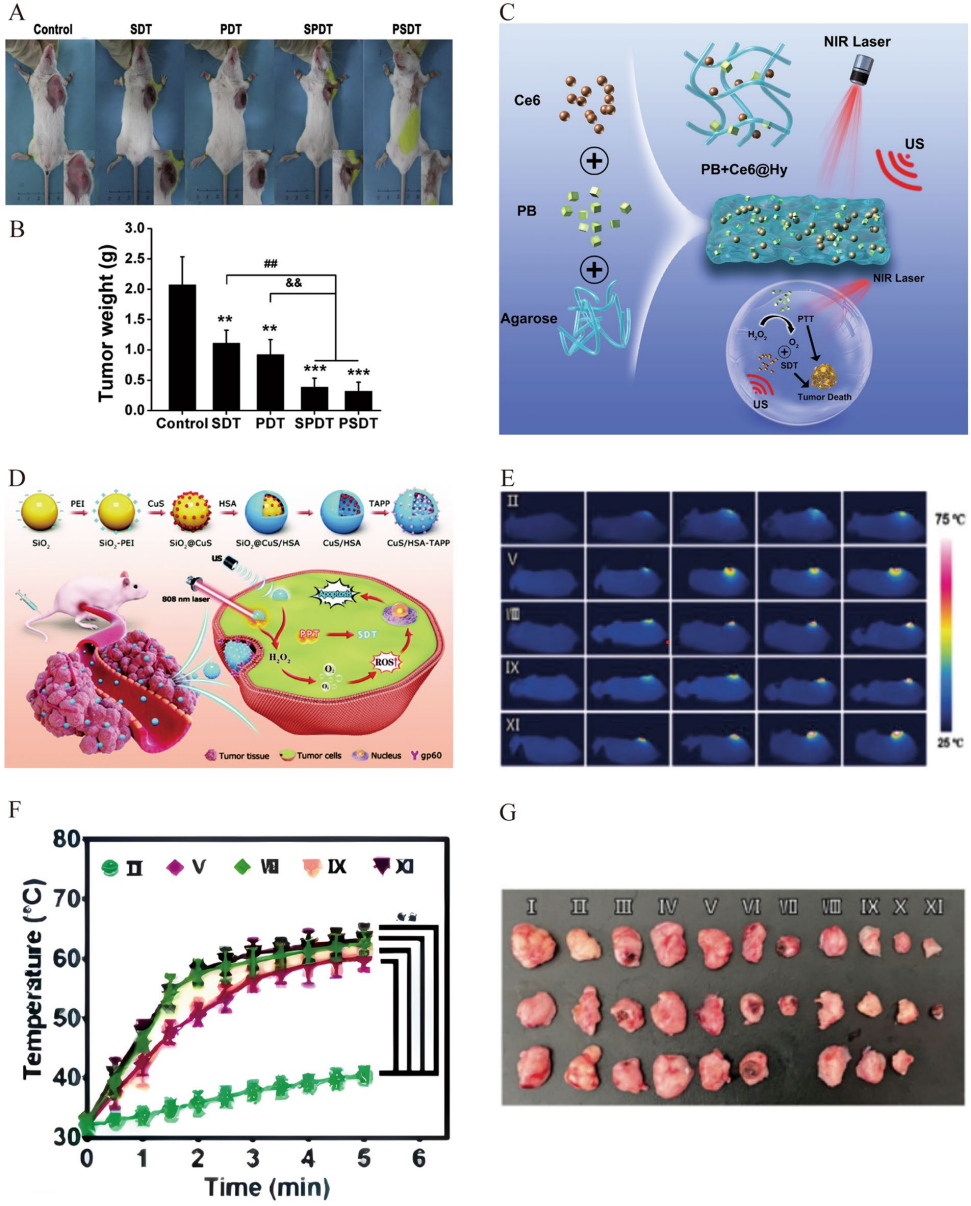
**A** Representative images of 4T1-bearing mice and **B** tumor weight from different groups at day 12 after treatments. ^**^P < 0.01 versus control, ^***^P < 0.001 versus control, ^##^*P* < 0.01 SPDT/PSDT group versus SDT group, ^&&^*P* < 0.01 SPDT/PSDT group versus PDT group. Reprinted with permission from ref. [108]. Copyright 2016, Elsevier. **C** Schematic illustration of PB + Ce6@Hy for self-augmented sonodynamic/photothermal combination therapy.Reprinted with permission from ref. [114]. Copyright 2022, Frontiers Media SA. **D** Schematic diagram of the preparation and anti-tumor mechanism of the CuS/HSA-TAPP. **E** Infrared thermal images and **F** temperature change curves of breast tumor-bearing mice with different treatments (**P < 0.01). **G** Tumor volume of breast tumor-bearing mice on the 14th day after different treatments. I: PBS group; II: Laser group; III:US group, IV: CuS/HSA hollow nanocapsules group, V: CuS/HSA hollow nanocapsules + laser group, VI: CuS/HSA hollow nanocapsules + US group, VII: CuS/HSA hollow nanocapsules + US + laser group, VIII: CuS/HSA-TAPP hollow nanocapsules group, IX: CuS/HSA-TAPP hollow nanocapsules + laser group, X: CuS/HSA-TAPP hollow nanocapsules + US group, XI: CuS/HSA-TAPP hollow nanocapsules + US + laser group. Reprinted with permission from ref. [115]. Copyright 2022, Royal Society of Chemistry

Wang et al. first reported SPDT treatment in three patients with metastatic breast cancer who did not respond to traditional therapy in clinical settings [14]. Sensitizer Sonoflora 1™ (SF1) was administered sublingually followed with 630 nm light (20 mWcm^−2^, 30 min) and US (1 MHz, 2.0 Wcm^−2^, 20 min) irradiation to perform SPDT. After treatment, the tumors and vital signs of these three patients improved to varying degrees, indicating that SPDT deserves further exploration for the treatment of breast cancer and its metastasis. Several studies have utilized Ce6 to achieve SPDT, which significantly inhibited primary tumor growth and lung metastasis in 4T1 tumor-bearing mice. They observed a decrease in matrix metalloproteinase and Bcl-2 expression, in addition to a significant increase in cleaved caspase-3 and PARP. Additionally, the ratio of LC3-II to LC3-I and beclin-1 increased, indicating that Ce6-SPDT induces mitochondria-dependent cell apoptosis and autophagy [105–107]. Liu et al. demonstrated that sinoporphyrin sodium (DVDMS) can serve as a sensitizer for SDT and PDT. In 4T1-bearing mice, the tumor weight inhibition rates of SDT (1.9 MHz, 1.6 Wcm^−2^, 3 min) and PDT (635 nm, 417 mWcm^−2^, 2 min, final light dose of 50 Jcm^−2^) alone were 46.66% and 55.54%, respectively. However, the tumor weight inhibition rates of SPDT and photodynamic and sonodynamic therapy were 81.45% and 84.83%, respectively, indicating that the effect was further enhanced when these two therapies were combined (Fig. 8A, B) [108]. This may be related to acoustic cavitation, chemical reactions, mechanical shearing, or ROS generation. In addition, the cell membrane permeability increased, which could improve drug uptake into tumor cells, exerting more effective anti-cancer effects. Previous studies have shown that a 150 J cm^−2^ dose of light can achieve satisfactory therapeutic effects when DVDMS is used for PDT [109]. This study achieved effective SPDT treatment by laser irradiation at only 50 Jcm^−2^. This may be because the subsequent SDT can compensate for the inevitable attenuation effect of PDT in deep tissues. The effectiveness of the combination therapy varies depending on the treatment sequence. PDT performed before SDT had a stronger therapeutic effect. A possible reason for this is that PDT has a higher dependence on the O_2_ concentration than SDT. However, owing to the complexity of SPDT systems, their mechanisms of action remain elusive. Further preclinical studies and clinical trials are required to validate and improve this new method.

### SDT combined with PTT

PTT can absorb light through photothermal agents and convert luminous energy into hyperthermia, prompting the thermal ablation of tumor cells [110]. PTT can alleviate hypoxia in tumor tissues and enhance the efficacy of oxygen-dependent SDT by increasing local blood circulation in tumor areas [111, 112]. In turn, ROS can strengthen the sensitivity of tumor cells to PTT. Accordingly, the combination of SDT and PTT can achieve competitive therapeutic effects and has enormous potential for various applications [112, 113].

Wang et al. encapsulated Prussian blue (PB) and Ce6 in a low-melting-point agarose hydrogel to construct PB + Ce6@Hy. In response to laser stimulation, the hydrogel gets heated and softened, promoting the release of Ce6 and PB into the tumor areas. PB exploits catalase activity to interact with endogenous H_2_O_2_ to generate extra O_2_ and promote the Ce6-mediated SDT effect, allowing the combined treatment of PTT and SDT to inhibit the growth of breast cancer (Fig. 8C) [114]. Tumor-targeted combination therapies have also been used to enhance the specificity of tumor treatment and reduce damage to normal tissues. Wang et al. constructed a novel hollow nanocapsule, CuS/HSA-TAPP, by co-loading tetra-(4-aminophenyl) porphyrin (TAPP) with hollow CuS/human serum albumin (HSA) nanocapsules (Fig. 8D) [115]. HSA was specifically recognized by gp60 overexpression in MCF-7 breast cancer cells, promoting targeted aggregation of CuS/HSA-TAPP in tumor cells. CuS/HSA exhibits photothermal conversion and peroxidase-like activity (Fig. 8E, F). Under laser irradiation, they improved tumor hypoxia and enhanced the synergistic effect of SDT and PTT in inhibiting MCF-7 tumor-bearing mice (Fig. 8G).

In summary, this combination therapy can utilize multifunctional sensitizers and photothermal effects to promote the targeted release and aggregation of sensitizers at tumor sites and overcome hypoxia in the TME to enhance SDT, which should be prioritised in future clinical translation.

### Ferroptosis-enhanced SDT

Breast cancer, particularly TNBC, has an inherent anti-apoptotic ability, which decreases the overall effectiveness of different treatments and the survival rate [116]. There is an urgent need to explore non-apoptotic therapies to counteract the anti-apoptotic effects in breast cancer cells. Ferroptosis is a type of programmed cell death mediated by LPO of polyunsaturated fatty acids (PUFAs) and the inhibition of GSH-dependent antioxidant mechanisms, such as the inhibition of a key regulator of ferroptosis, GPX4 [117]. Nevertheless, ferroptosis has not yet achieved ideal prospects for tumor treatment because of the limitations of low intracellular ROS levels. SDT exerts tumor cell-killing effects by generating intracellular ROS and may have the potential to improve ROS-mediated ferroptosis. Additionally, tumor cells inhibit ROS-mediated oxidative stress by negatively regulating ferroptosis. Thus, activating ferroptosis can effectively increase the sensitivity of tumor cells to ROS [118], indicating that SDT may be an ideal combination therapy for ferroptosis.

**Fig. 9:**
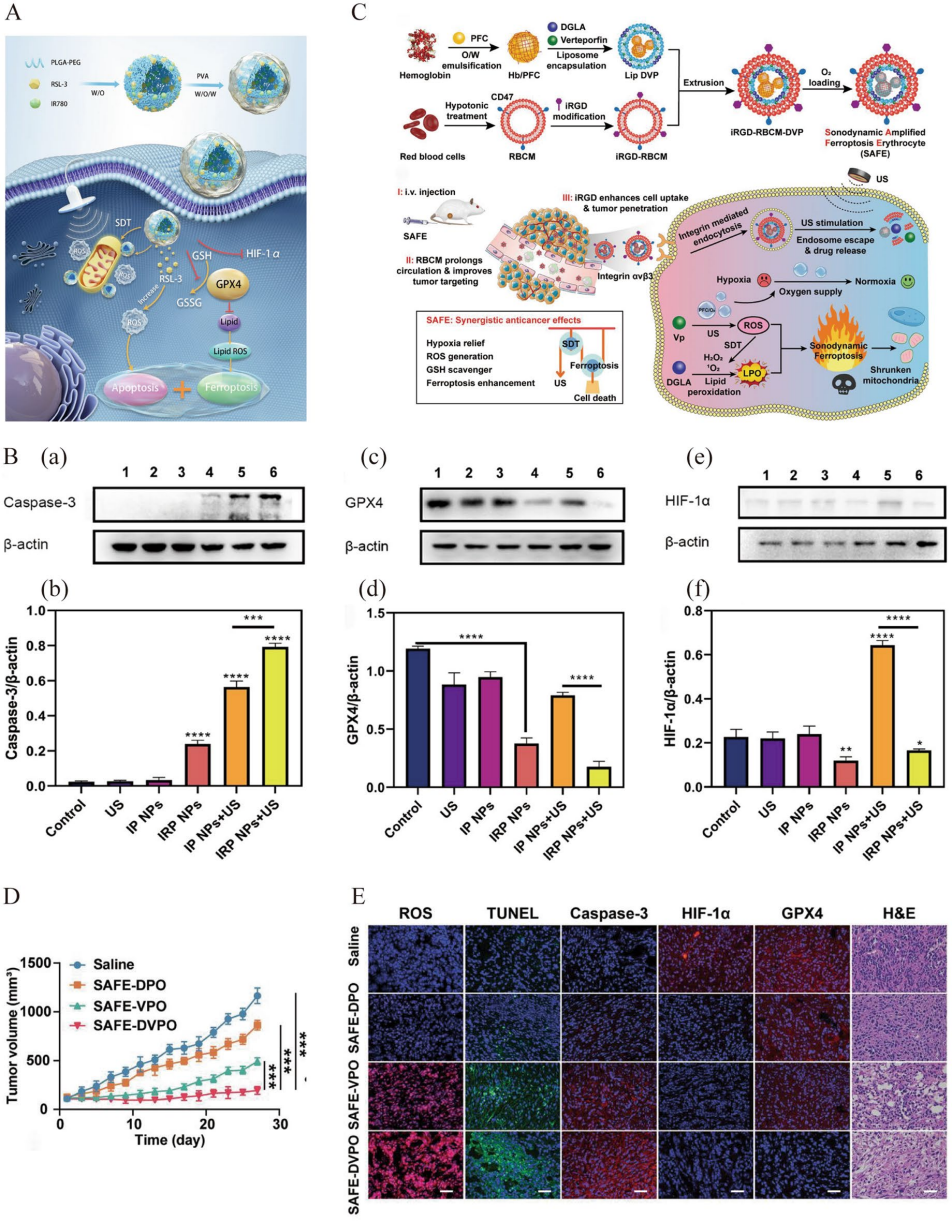
**A** Schematic illustration of IRP NPs for ferroptosis-boosted sonodynamic antitumor therapy. **B** Western blot and relative quantitative analysis of (a and b) Caspase-3, (c and d) GPX4 and (e and f) HIF-1α (^**^P < 0.01, ^***^P < 0.001, ^****^P < 0 .0001). Reprinted with permission from ref. [119]. Copyright 2022, Taylor &Francis. **C** Schematic diagram of SAFE mediated combination treatment of sonodynamic therapy and ferroptosis. **D** The tumor volumes curves of the 4T1 orthotopic breast tumors with different treatments (***P < 0.001). **E** Histological analysis of tumor tissues for ROS, TUNEL, caspase-3, HIF-1α and GPX4. Reprinted with permission from ref. [120]. Copyright 2022, Wiley

Wang et al. constructed a new mitochondria-targeting nanosystem, named IRP NPs, by loading IR780 and the GPX4 inhibitor Ras-selective lethal 3 (RSL3) into PLGA in combination with ferroptosis and SDT to enhance anti-tumor efficacy (Fig. 9A) [119]. IR780 preferentially aggregates in the mitochondria, mediating mitochondria-targeted SDT. Treatment with IRP NPs and US (3 Wcm^−2^, on 2 min, off 2 min, two cycles) showed the most significant tumor growth inhibition in 4T1-bearing mice, which was related to the highest ROS generation, a more significant decrease in the MMP, and disruption of mitochondrial membrane integrity. Mechanism studies revealed that RSL-3 inhibited GPX4 and reversed SDT-induced hypoxia-inducible factor-1 alpha (HIF-1α) upregulation to amplify ROS production, promoting ferroptosis and SDT-mediated cell apoptosis to kill tumors synergistically (Fig. 9B). Zhou et al. designed an engineered erythrocyte named sonodynamic amplified ferroptosis erythrocyte (SAFE) for synergistic enhancement of SDT and ferroptosis via assembling hemoglobin (Hb), PFC, a ferroptosis activator (dihomo-γ-linolenic acid, DGLA), and a sonosensitizer (verteporfin, Vp) into a mixed camouflage of internalized RGD peptide (iRGD) and red blood cell membrane (RBCM) (Fig. 9C) [120]. The O_2_ supply through the Hb/PFC complexes promotes Vp-mediated SDT in response to US stimulation. DGLA and the ROS produced during SDT can activate ferroptosis to synergistically inhibit tumor growth. In addition, RBCM modification prolonged its circulation time, whereas iRGD peptide modification allowed it to accumulate preferentially in tumor tissues, improving its anti-cancer efficacy and minimizing the side effects. Under US stimulation (1 MHz, 2 Wcm^−2^, 3 min), the SAFE-containing DGLA, Vp, and oxygen-rich PFC (SAFE-DVPO) treatment group had the strongest ROS generation ability and tumor growth inhibition effect in 4T1-bearing mice (Fig. 9D). SAFE-DVPO alleviated hypoxia in tumor tissues and effectively enhanced ferroptosis through the combined effects of ROS production triggered by SDT and lipid peroxidation mediated by DGLA to kill tumor cells (Fig. 9E).

To overcome the limitation of finite supply of PUFAs on ferroptosis sensitivity, Wu et al. constructed 4T1 cells with high expression of acyl-CoA synthetase long-chain family member 4 (ACSL4) and catalase (Cat) through lentiviral transfection. Then exosomes enriched in Cat and ACSL4 (EXO@CA) were extracted and conjugated with sonosensitizer tetrakis (4-carboxyphenyl) porphyrin (TCPP) via electroporation to produce engineered exosomes (EXO@CAT), providing a new insight into the effective synergistic effect between ferroptosis and SDT [121]. Under US stimulation, Cat in EXO@CAT can react with H_2_O_2_ inside the tumor to improve the hypoxia of the TME, enhancing the ROS generation and promoting the accumulation of LPO, ultimately leading to ferroptosis. Meanwhile, ACSL4, an essential enzyme which converts free PUFAs into membrane phospholipids for LPO to enhance the sensitivity of tumor cells to ferroptosis, also increases LPO accumulation.

In conclusion, the establishment of nanoplatforms combining ferroptosis activators and novel sonosensitizers is a prospective method for apoptosis resistant breast cancer.

### SDT combined with chemodynamic therapy (CDT)

CDT utilizes endogenous H_2_O_2_ in the TME to generate ·OH through Fenton or Fenton-like reactions catalyzed by nanomaterials containing transition metals [122]. However, insufficient H_2_O_2_ within the TME limits the Fenton reaction rate, which is the main obstacle to CDT [123]. Thus, doping transitional metal ion in inorganic sonosensitizers can effectively promote the separation efficiency of e^−^ and h^+^ and increase ROS generation upon US irradiation. Under intratumoral excess H_2_O_2_ condition and US irradiation, the newly designed multifunctional sonosensitizer doped with transitional metal ion can achieve a combination therapy of CDT and SDT for anti-tumor treatment by generating more ROS.

**Fig. 10:**
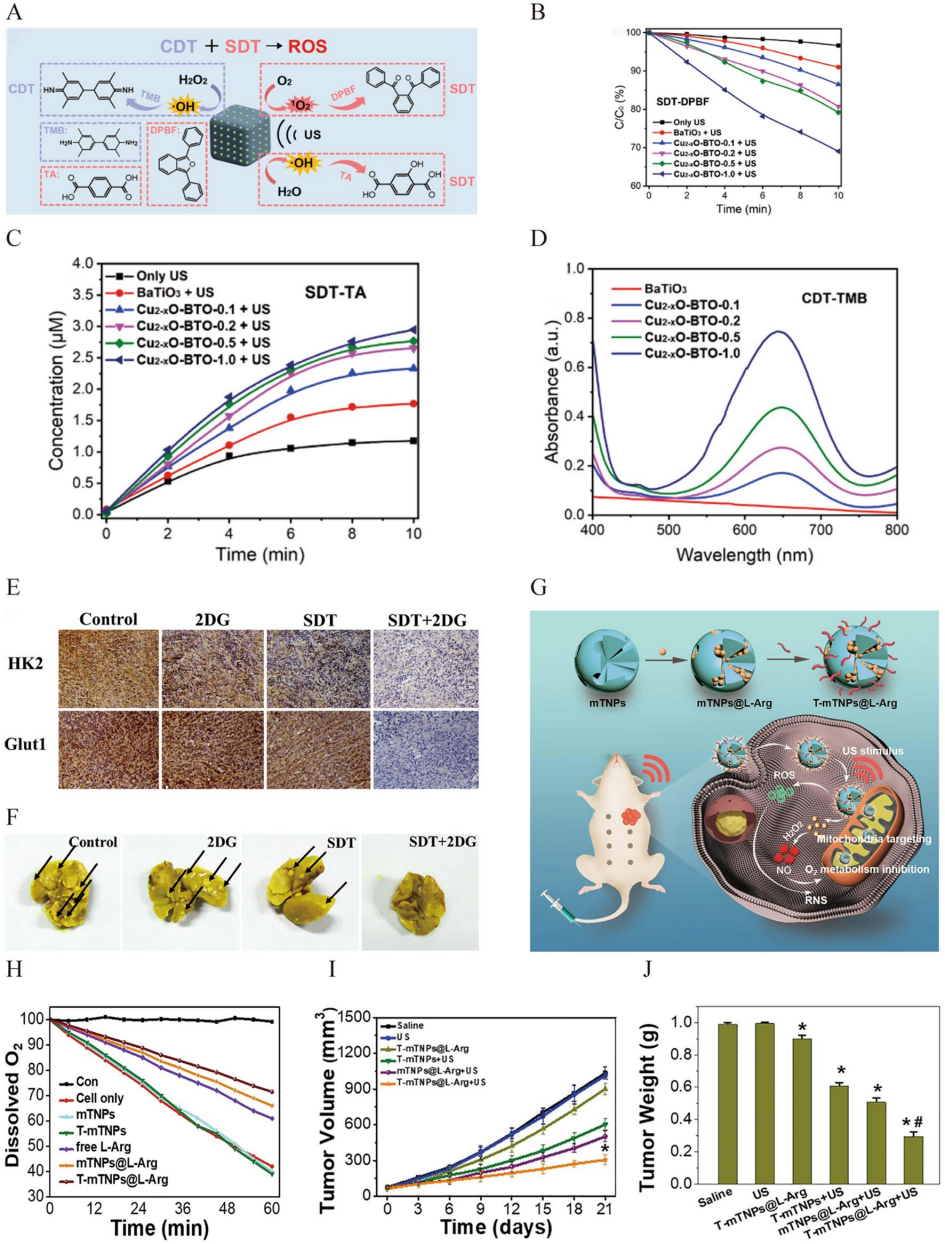
**A** Schematic diagram of ROS generation by Cu_2_-_x_O-BTO NCs through the sonodynamic and chemodynamic processes and detection by the specific probes. **B** The detection of ^1^O_2_ in the sonodynamic process via degradation curves of DPBF. **C** The production of ⋅OH in the sonodynamic process measured by using TA as the probe. **D** Generation of ⋅OH in the chemodynamic process measured by using TMB as the probe. Reprinted with permission from ref. [124]. Copyright 2022, American Chemical Society. **E** The expression of HK2 and Glut1 in 4T1 tumor-bearing mice by immunochemistry. **F** Images of pulmonary metastatic nodules of breast cancer. Reprinted with permission from ref. [131]. Copyright 2019, Elsevier. **G** Schematic illustration of T-mTNPs@L-Arg for synergistic nitric oxide gas-sonodynamic therapy. **H** The relative dissolved oxygen content in the cell medium of different groups. **I** The tumor volumes curves (*P < 0.05 versus mTNPs@L-Arg) and **J** Tumor weight of the MCF-7 tumor-bearing mice with different treatments (*P < 0.05 versus the control groups, #P < 0.05 versus mTNPs@L-Arg). Reprinted with permission from ref. [132]. Copyright 2022, Dove Press

Zhao et al. achieved synergistic treatment in breast cancer with SDT and CDT using Cu_2_-_x_O-BaTiO_3_ nanocubes (Cu_2−x_O-BTO NCs) (Fig. 10A) [124].The heterojunction of Cu_2−x_O-BTO promoted the separation and migration of e^−^ and h^+^, greatly enhancing its ROS generation ability. Owing to the presence of Cu (I), Cu_2_-_x_O-BTO could undergo the Fenton reaction with local H_2_O_2_ in the TME to generate ·OH, allowing CDT to directly kill tumor cells (Fig. 10B–D). Cu_2−x_O-BTO and US (1.0 MHz, 1.0 Wcm^−2^, 5 min, 50% duty cycle) had the highest tumor inhibition effect on 4T1-bearing mice, suggesting that Cu_2−x_O-BTO, as a sonosensitizer and Fenton-like agent, could promote the combined action of SDT and CDT to inhibit breast cancer. Similarly, Chen et al. constructed a hollow TiO_2_ nanosonosensitizer doped with single-atom copper (Cu/TiO_2_) to synergistically enhance sonodynamic and chemodynamic therapeutic effects against TNBC [125]. The percentage of apoptotic 4T1 cells in the Cu/TiO_2_ and US (1.0 Wcm^−2^, 1 MHz, 50% duty cycle, 5 min) group was 99.4%, which was much higher than that in the TiO_2_ and US group (86%). In addition, the inhibition rate of the Cu/TiO_2_ and US treatment of breast tumors in 4T1-bearing mice was the highest at 70.35%, particularly compared to the TiO_2_ and US group (30.91%). The potential mechanism underlying the synergistic effects of Cu/TiO_2_ was further investigated. It was found that ROS generated by the synergistic therapy could cause extensive DNA damage, which could activate the p53 signalling pathway to induce apoptosis. In addition, ROS-dependent inhibition of oxidative phosphorylation occurs, thereby inducing an energy crises and apoptosis of cancer cells. Meanwhile, Cu/TiO_2_ could increase mitochondrial permeability and promote ROS production through the action of TNF, leading to cancer cell apoptosis.

Considering the impact of diverse cell types in the TME on the enrichment efficiency of sonosensitivers in tumor tissues, Zhang et al. designed dual-targeting biomimetic semiconducting polymer nanocomposites (SPFeNOC) using osteoclasts and 4T1 breast cancer cells. The core structure consists of semiconducting polymer and iron oxide (Fe_3_O_4_) nanoparticles, whereas, the surface is coated with hybrid membrane derived from osteoclasts and 4T1 breast cancer cells. This hybrid membrane facilitates targeted delivery to metastatic tumor cells and osteoclasts in bone metastases through a homologous targeting mechanism, thereby enhancing nanoparticle accumulation in tumors. The semiconducting polymer enables NIR fluorescence imaging and SDT, whereas Fe_3_O_4_ nanoparticles serve as contrast agents for MRI and mediate CDT. This combined SDT-CDT approach effectively inhibits breast cancer bone metastases [126].

In conclusion, this study provides a good example of non-invasive nanocatalytic medicine for synergistic breast cancer treatment.

### Others

Glycolysis in tumor cells is increased to meet the need for abnormal growth, which can facilitate the proliferation and metastasis of tumor cells. Targeted inhibition of glycolysis to achieve starvation therapy in tumor cells may be an effective approach for inducing cancer cell death [127]. The most commonly used antiglycolytic drug is 2-deoxyglucose (2-DG), which can inhibit the energy supply, increasing the sensitivity of tumor tissue to various therapies, such as chemotherapy and PDT [128, 129]. Similar to PDT, SDT exerts anti-tumor effects by activating sonosensitizers to produce ROS upon US irradiation. Therefore, the combination of 2-DG and SDT is expected to enhance the therapeutic efficacy in breast cancer. Xie et al. utilized DVDMS and 2-DG to realize mitochondrial SDT and starvation therapy [130], which remarkably promoted cell apoptosis and inhibited tumor growth in 4T1-bearing mice by increasing ROS generation, reducing the MMP, and inhibiting oxidative phosphorylation to supply energy. A similar combination therapy scheme showed that the growth of primary and lung metastasis of breast cancer was inhibited by blocking cell cycle progression and the expression of the key limiting enzymes hexokinase II (HK2) and glucose transporter 1 (Glut1) in the lycolytic pathway, as well as their coupling with mitochondria (Fig. 10E) [131]. These results indicate that co-therapy is a promising method for inhibiting the proliferation and metastasis of breast cancer (Fig. 10F).

Nitric oxide (NO) has been found to have multiple physiological and pathological activities, including consuming intracellular GSH, triggering NO poisoning and reacting with ROS to produce more lethal reactive nitrogen species (RNS). Further, NO can remarkably decrease the O_2_ consumption of tumor cells and alleviate the limitation of hypoxia on the ROS generation of SDT by suppressing mitochondrial complex IV, a key enzyme for the electron transport chain of mitochondrial respiration. Thus, the combination of SDT and gas therapy to generate NO is expected to be an efficient cancer therapy due to their synergistic effects. Zuo et al. constructed a multifunctional nanoplatform named T-mTNPs@l-Arg by combining the NO donor l-arginine (l-Arg) and mesoporous titanium dioxide nanoparticles (mTNPs) with the mitochondria-targeting ligand triphenylphosphonium (TPP). T-mTNPs@l-Arg achieved mitochondria-targeted SDT in combination with NO gas therapy (Fig. 10G) [132]. NO can exert synergistic effects with SDT by inhibiting mitochondrial complex IV to reduce cellular O_2_ consumption and by reacting with ROS to produce more toxic RNS (Fig. 10H) [133]. The T-mTNPs@l-Arg and US (1 MHz, 1 Wcm^−2^, 1 min) group also showed the most significant inhibitory effect on tumor volume and weight in MCF7-bearing mice (Fig. 10I, J, Table 6).

**Table 6:** Summary of SDT-based sonosensitizers for combination therapy of breast cancer

Therapies	Sonosensitizers	Model	US power	References
SDT + chemotherapy	Pp18-lipos@SRA737&DOX	MDA-MB-231		[17]
	AS1411-DOX/PFH-PEG@PLGA	4T1	80 Wcm^−2^, 5 s	[18]
	EV (TPP-Ce 6/PL)	MCF-7	1 MHz, 0.3 Wcm^−2^, 3 min	[134]
	DH-HM@BSA	4T1	1 MHz, 1.5 Wcm^−2^, 3 min	[60]
	T80(T-Ce6/PL)	MCF-7	1 MHz, 0.3 Wcm^−2^, 2 min	[135]
	LDH-MTX@CM_M_-Ce6	MCF-7	1 MHz, 1 Wcm^−2^, 4 min	[136]
	DOXSFNPs	4T1	1 MHz, 1 Wcm^−2^, 4 min	[137]
	mTSeIR	MDA-MB-231	3 Hz, 3 Wcm^−2^, 5 min	[138]
SDT + immunotherapy	CCL21a/Exo^GM−CSF+Ce6^@nanoGel	4T1	1 MHz, 2 Wcm^−2^,5 min	[94]
	SnSNP	4T1	1 MHz, 2 Wcm^−2^	[97]
	CPDA@PFH	4T1		[139]
	IDP@O_2_	4T1	1 MHz, 1.6 Wcm^−2^, 8 min	[140]
	CCLT@FT	4T1	1 MHz, 1 Wcm^−2^, 5 min	[141]
	RmPLH	4T1	1 MHz, 1.5 Wcm^−2^, 2 min	[142]
	PATCM	4T1	1 Wcm^−2^, 10 min	[143]
	G5-CHC-R	4T1	1 MHz, 2 Wcm^−2^, 20 min	[144]
	LP@PFH@HMME	4T1	1 MHz, 1.6 Wcm^−2^, 5 min	[145]
	GdNPs/Ce6-Ric	4T1	1 MHz, 1.5 Wcm^−2^, 5 min	[146]
SDT + PDT	Ce6	MDA-MB-231	1 MHz, 0.36 Wcm^−2^, 1 min	[106]
	Ce6	4T1	1.9 MHz, 1.6Wcm^−2^, 3 min	[105]
	Ce6	4T1	1 MHz, 1 min	[107]
	DVDMS	4T1	1.9 MHz, 1.6 Wcm^−2^, 3 min	[108]
	Bi_2_S_3-x_-Au@HA	4T1	50 kHz, 3.0 Wcm^−2^, 5 min	[147]
	Chl-MOF	4T1	1 MHz, 1.0 Wcm^−2^, 2 min	[72]
SDT + PTT	PPBP-B-TiO_2_	4T1	1 Wcm^−2^, 3 min	[148]
	Ce6	4T1	3 MHz, 1 Wcm^−2^, 40 s	[114]
	CuS/HSA-TAPP	MCF-7	1 MHz, 1 Wcm^−2^, 2 min	[115]
	HCuS@Cu2S@Au–P(NIPAM-*co*-AAm)-PpIX Nanohybrids	MDA-MB-231	3 MHz	[149]
Ferroptosis-enhanced SDT	IRP NP	4T1	3 Wcm^−2^	[119]
	SAFE-DVPO	4T1	1 MHz, 0.35Wcm^−2^	[120]
	A-UIO-66-CoO_x_	4T1	1 MHz, 1.5 Wcm^−2^, 5 min	[150]
	TiF NPs	4T1	1 MHz, 2 Wcm^−2^, 10 min	[151]
SDT + CDT	Cu_2_-_x_O-BTO NC	4T1	1 MHz, 1 Wcm^−2^,5 min	[124]
	Cu/TiO_2_	4T1	1 MHz, 1 Wcm^−2^,5 min	[125]
	BOC-Fe NSs	4T1	50 kHz, 1 Wcm^−2^, 3 min	[152]
Others	DVDMS	4T1	1.9 MHz, 2Wcm^−2^	[130]
	DVDMS	4T1	2W, 2 min	[131]
	FC-COS/β-CD-TPP/GOxNPs	4T1	2 MHz, 1 Wcm^−2^, 10 min	[153]
	T-mTNPs@L-Arg	MCF-7	1 MHz, 1 Wcm^−2^, 1 min	[132]
	TAM@BP-FA	MCF-7	1 MHz, 1.5 Wcm^−2^, 3 min	[154]
	BP@Cu_2_O@L-Arg	4T1	1.5 Wcm^−2^, 3 min	[155]
	Bi/BiVO_4_	4T1	1 MHz, 0.5 Wcm^−2^, 10 min	[156]

## Conclusion and perspectives

As a promising alternative to conventional therapies, SDT offers unique advantages in breast cancer treatment. Its efficacy relies on US stimulation of sonosensitizers to generate cytotoxic ROS, effectively targeting and eliminating tumor cells. Compared to traditional therapies such as surgery, chemotherapy, and radiotherapy, SDT is non-invasive, has minimal side effects, and exhibits lower systemic toxicity. Despite its notable clinical potential, developing highly efficient sonosensitizers to enhance ROS remains a critical step in maximizing SDT’s effectiveness. Conventional sonosensitizers face challenges in clinical translation owing to issues with stability, poor tumor accumulation, and low bioavailability [157, 158]. However, the unique properties of nanomaterials, such as the high surface area-to-volume ratio and tunable surface characteristics have enabled the development of multifunctional nanosonosensitizers or nanoplatforms [159, 160]. By loading appropriate agents and functionalizing with various ligands, these platforms offer new strategies to improve ROS-based SDT for breast cancer ablation. Furthermore, nanomaterials have facilitated synergistic effects between SDT and other therapies, including sono-chemotherapy, sono-immunotherapy, and sono-photodynamic therapy, by encapsulating therapeutic drugs to target primary breast cancer as well as distal and metastatic sites.

Although SDT has shown notable promise in the treatment of breast cancer, several challenges must be addressed to facilitate its clinical translation:

1. Enhancing the therapeutic efficiency and selectivity of SDT requires the development of novel sonosensitizers, particularly multifunctional ones [161]. Commonly used organic sensitizers often suffer from low water solubility, poor chemical stability, and complex synthesis processes. Inorganic sensitizers, on the other hand, face challenges related to low biocompatibility, limited biodegradability and inadequate accumulation in cancer cells. Additionally, their tendency to rapidly combine with e^−^ and h^+^ limits ROS generation. Thus, the synthesis of novel sonosensitizers with high ROS yield, improved stability, better tumor targeting, biocompatibility, and biodegradability is essential for advancing SDT efficacy [162].
2. Targeted delivery of sonosensitizers to tumor tissues without inducing cytotoxic effects on healthy cells is essential for advancing their clinical translation. Furthermore, these molecules must be efficiently cleared from the body. Targeted delivery can be achieved by encapsulating sonosensitizers within various carriers such as nanoparticles, liposomes, and mesoporous silica. However, inorganic and synthetic carriers may present inherent toxicity and immunogenicity, which can vary depending on the dosage. In contrast, extracellular vesicles (EVs) and cell membrane-coated biomimetic nanomaterials show promise as clinically applicable nanocarriers, offering targeted delivery to tumor sites with minimal or no immunogenicity [57, 126, 134, 138, 143, 163–165].
  For example, Yang et al. developed a nontoxic microcystin strain as a novel reagent for SDT. Under red light irradiation, this strain not only produces O_2_ but also utilizes its vesicles and phycocyanin as cavitation nuclei and sonosensitizers, respectively. Upon US irradiation, it mediates the SDT effect, releasing algal fragments that activate the Toll-like receptor pathway, triggering a cascade of immune responses [166]. Additionally, a hybrid membrane (HM) was constructed by fusing breast cancer cell membranes with bacterial outer membrane vesicles. IR780-loaded PLGA was then encapsulated with the HM to form nanoparticles, IR780@PLGA@HM, which exhibited good biocompatibility and effectively targeted 4T1 tumors. These nanoparticles exert sonodynamic immunity and demonstrate strong therapeutic efficacy in treating breast cancer bone metastasis [93]. The inherent tendency of bacteria to accumulate in hypoxic areas and their ability to penetrate the complex TME offer new directions for targeted delivery and accumulation of therapeutic agents in tumor tissues. Probiotics, as a representative biological carrier, have low in vivo toxicity. Coupling multifunctional nanoparticles onto these carriers can achieve targeted and safe anti-tumor effects [167].
3. It is necessary to optimize the combination of drug/sensitizers and US parameters. The therapeutic effect of SDT is influenced by US parameters, especially intensity, frequency, and stimulation duration, since the implementation of SDT requires US to activate sonosensitizers [168]. Nevertheless, there is uncertainty in US parameters during SDT, which can affect study replication and limit the generalizability of research findings owing to the lack of standardized or widely used US equipment for SDT. Wang et al. developed a specialized US system with precise US parameter description and a step-by-step sonodynamic strategy for activating phytochlorin-based sonosensitizers, transitioning from in vitro to in vivo applications. This consistent US output is reproducible and can be extensively implemented by other researchers, providing a strong framework for in vitro and in vivo applications of sonodynamic techniques and facilitating the advancement of SDT in clinical trials [169]. Furthermore, the development of wearable US systems offers promising strategies for the creation of portable, personalized anti-tumor treatment schemes in clinical practice [170]. Therefore, extensive additional preclinical trials can utilize specialized ultrasound equipment to optimize tumor-specific US parameters and promote the generalizability of related researches [171].
4. Combining the heterogeneity, molecular phenotype and mechanisms of therapeutic resistance of different pathological types of breast cancer, it is necessary to explore more complementary combination therapies, such as gene therapy or microbial-based therapy, to provide comprehensive treatment options for breast cancer [172, 173]. Gene therapy, represented by small interfering RNA (siRNA), has developed into an effective cancer treatment method by utilizing exogenous nucleic acids to upregulate or downregulate target genes, thus inhibiting tumor invasion and proliferation. However, the nonspecific distribution of siRNA poses challenges in achieving efficient accumulation in tumor tissues. The use of nanocarriers such as nanobubbles (NBs) to deliver siRNA and sonosensitizers can improve gene transfection efficiency through US-targeted nanobubble disruption, enabling the combined use of gene therapy and sonodynamic therapy. He et al. employed NBs to co-load siTRIM37 and IR780, facilitating the combination of SDT and gene therapy against TNBC. US irradiation induces the rupture of siRNA@IR780 NBs rupture, promoting the entry of siTRIM37 and IR780 into cells and inhibiting TRIM37 expression [174].
5. Further research should be conducted on the mechanisms of SDT and SDT-based combination therapies for breast cancer treatment. It has been verified that ROS-mediated oxidative stress is the main cause of cancer cell death through SDT. Nevertheless, it is still unknown whether other cancer cell death paths are involved in addition to necrosis, apoptosis, or ferroptosis. For instance, pyroptosis, unlike apoptosis, can trigger a strong anti-tumor immune response [175–177]. Therefore, converting cell apoptosis induced by SDT to pyroptosis could enhance the immunogenicity of SDT. Wang et al. proposed a new strategy to enhance SDT by epigenetically induced pyroptosis using a nanocoordinator (HTA) constructed through metal-phenolic coordination involving TiO_2_ nanoparticles, Aza (a DNA methyltransferase inhibitor), and polyphenol-modified hyaluronic acid. When Aza restores GSDME expression, TiO_2_ produces ROS under US stimulation, activating caspase-3 and inducing pyroptosis through Gasdermin E cleavage. HTA improved anti-tumor immunity and enhanced the efficacy of SDT in an orthotopic breast cancer model [178]. Thus, exploring the pathways of SDT induced cell death can not only guide the design of novel sonosensitizers targeting specific cell death pathways, but also contribute to more precise SDT-based synergistic therapies, potentially expanding the application scope of SDT in oncological treatments.

In recent years, there has been an increase in the number of SDT undergoing clinical trials. As of January 2025, Clinical trials.gov lists 13 trials filtered for the search term “sonodynamic therapy” (Table 4). The majority applications of these clinical trials are focused on brain tumor treatments, including high-grade glioma, recurrent glioblastoma, diffuse intrinsic pontine gliomas, diffuse midline gliomas, cholangiocarcinoma, and atherosclerosis. The first human phase 0 clinical trial (NCT04559685) reported that ALA can be safely used for SDT treatment of recurrent high-grade glioma without off-target cell effects. And the dose escalation and expansion study are currently ongoing. These human clinical trials reveal that SDT is safe and has the potential to treat tumors. It is believed that with the joint development of medicine, biology, and nanomaterials, more ideal nanosonosensitizers and synergistic therapies will be designed to provide a safer and more effective option for patients with breast cancer [164, 179, 180] (Table 7).

**Table 7:** Clinical trials on SDT

Sonosensitizers	Application	Study title	Study type	NCT number	Phase
5-ALA	Diffuse intrinsic pontine glioma and diffuse midline glioma	A phase 2 study of sonodynamic therapy using SONALA-001 and Exablate 4000 type 2.0 in patients with DIPG	Interventional	NCT05123534	Phase 2
5-ALA	Cerebral glioblastomas	Sonodynamic therapy with ExAblate system in glioblastoma patients	Interventional	NCT04845919	Phase 2
5-ALA	High-grade glioma	Clinical trial evaluating safety of 5-aminolevulinic acid (5-ALA) combined with CV01 delivery of ultrasound for sonodynamic therapy (SDT) in patients with newly diagnosed high-grade glioma (HGG) prior to resection and standard adjuvant therapy (ALA SDT GLIOMA 401)	Interventional	NCT06665724	Phase 1
5-ALA	Recurrent glioblastoma	Sonodynamic therapy in patients with recurrent GBM	Interventional	NCT06039709	Phase 1
Hematoporphyrin	Cholangiocarcinoma	An exploratory clinical study of photodynamic therapy combined with sonodynamic therapy in cholangiocarcinoma	Interventional	NCT05580328	Not applicable
ALA	High-grade glioma	Study of sonodynamic therapy in participants with recurrent high-grade glioma	Interventional	NCT04559685	Early Phase 1
ALA	Recurrent glioblastoma	A study of sonodynamic therapy using SONALA-001 and Exablate 4000 type 2.0 in subjects with recurrent GBM	Interventional	NCT05370508	Phase 1/2
5-ALA	High-grade glioma	Study to evaluate 5-ALA combined with CV01 delivery of ultrasound in recurrent high-grade glioma	Interventional	NCT05362409	Phase 1
Sinoporphyrin sodium	Atherosclerosis	Sonodynamic therapy manipulates atherosclerosis regression trial on patients with PAD and claudication	Interventional	NCT03457662	Phase 1/2
Sinoporphyrin sodium	Atherosclerosis	Sonodynamic therapy manipulates atherosclerosis regression trial on patients with carotid atherosclerotic plaques	Interventional	NCT03382249	Phase 1/2
Sinoporphyrin sodium	Atherosclerosis	Sonodynamic therapy on patients with femoropopliteal PAD and claudication	Interventional	NCT03318484	Phase 1/2
Sinoporphyrin sodium	Atherosclerosis	Sonodynamic therapy in the treatment of carotid atherosclerosis (SMART-C)	Interventional	NCT03871725	Phase 1
Sinoporphyrin sodium	Peripheral arterial disease	Sonodynamic therapy in the treatment of perivascular adipose tissue on patients with PAD and claudication	Interventional	NCT03967730	Phase 1/2

## Data Availability

No datasets were generated or analysed during the current study.

## Abbreviations

SDT: Sonodynamic therapy
US: Ultrasound
ROS: Reactive oxygen species
e^−^: Electrons
h^+^: Holes
GSH: Glutathione
TME: Tumor microenvironment
O_2_: Oxygen
·O_2_^−^: Superoxide anions
^1^O_2_: Singlet oxygen
·OH: Hydroxyl radicals
PpIX: Protoporphyrin IX
5-ALA: 5-Aminolevulinic acid
Ce6: Chlorin e6
TiO_2_: Titanium dioxide
MMP: Mitochondrial membrane potential
HCQ: Hydroxychloroquine
LPO: Lipid peroxidation
GPX4: Glutathione peroxidase 4
MOFs: Metal–organic frameworks
2D: Two-dimensional
MOL: Metal–organic layer
TBP: 5,10,15,20-Tetra(*p*-benzoato)porphyrin
ACQ: Aggregation-induced quenching
TTET: Triplet–triplet energy transfer
3D: Three-dimensional
COF: Covalent organic framework
PTT: Photothermal therapy
BiOCl NSs: Bismuth oxychloride nanosheets
SP: Soybean phospholipids
H_2_O_2_: Hydrogen peroxide
1-Zn-PPA: 1-Zn-chelated pheophorbide a
cRGD: Cyclic arginine–glycine–aspartate
PDT: Photodynamic therapy
ICD: Immunogenic cell death
IDO1: Indoleamine 2,3-dioxygenase 1
CTL: Cytotoxic T lymphocyte
ZIF-8: Zeolitic imidazole frameworks-8
BP: Black phosphorus
G6PD: Glucose-6-phosphate dehydrogenase
ICG: Indocyanine green
PFC: Perfluorocarbon
PLGA: Polylactic-*co*-glycolic acid
PFTBA: Perfluorotributylamine
DOX: Doxorubicin
MNPs: Melanin nanoparticles
PA: Photoacoustic imaging
FA: Folate
HMME: Hematoporphyrin monomethyl ether
PFP: Perfluoropentane
DTX: Docetaxel
CEUS: Contrast-enhanced ultrasound
MONs: Mesoporous organosilica nanoparticles
MRI: Magnetic resonance imaging
PEG: Polyethylene glycol
Pp18-lipos: Liposome-like nanoporphyrin
PFH: Perfluorohexane
TNBC: Triple-negative breast cancer
ICB: Immune checkpoint blockade
GM-CSF: Granulocyte–macrophage colony-stimulating factor
DCs: Dendritic cells
IL-2: Interleukin-2
IFN-γ: Interferon-γ
TNF-α: Tumor necrosis factor-α
TGF-β: Transforming growth factor-β
ECM: Extracellular matrix
NIR: Near-infrared
SnSNPs: Tin monosulfide nanoparticles
GcMAF: Gc protein-derived macrophage-activating factor
FDA: Food and Drug Administration
DMSNs: Dendritic mesoporous silica nanoparticles
PD-L1: Programmed death-ligand 1
aPD-L1: Programmed death-ligand 1inhibitor
SPDT: Sonodynamic and photodynamic therapy
SF1: Sonoflora 1™
DVDMS: Sinoporphyrin sodium
PB: Prussian blue
TAPP: Tetra-(4-aminophenyl) porphyrin
HSA: Human serum albumin
RSL3: Ras-selective lethal 3
HIF-1α: Hypoxia-inducible factor-1 alpha
SAFE: Sonodynamic amplified ferroptosis erythrocyte
Hb: Hemoglobin
DGLA: Dihomo-γ-linolenic acid
Vp: Verteporfin
iRGD: Internalized RGD
RBCM: Red blood cell membrane
ACSL4: Acyl-CoA synthetase long-chain family member 4
Cat: Catalase
EXO@CA: Exosomes enriched in Cat and ACSL4
TCPP: Tetrakis (4-carboxyphenyl) porphyrin
EXO@CAT: (EXO@CA) conjugated with TCPP
SPFeNOC: Semiconducting polymer nanocomposites
Fe_3_O_4_: Iron oxide
CDT: Chemodynamic therapy
2-DG: 2-Deoxyglucose
HK2: Hexokinase II
Glut1: Glucose transporter 1
RNS: Reactive nitrogen species
NO: Nitric oxide
l-Arg: L-arginine
mTNPs: Mesoporous titanium dioxide nanoparticles
TPP: Triphenylphosphonium
DH-HM@BSA: DOX/HMME-HMnO_2_@bovine serum albumin (BSA)
T80 (T-Ce6/PL): Tween 80 micelles encapsulated with piperlongumine and TPP-conjugated Ce6
LDH-MTX@CM_M_-Ce6: Methotrexate-loaded layered double hydroxide coated with chlorin e6-modified MCF-7 cell membranes
mTSeIR: Diselenide-conjugated sonosensitizers and tirapazamine, encapsulated within M1 macrophage membrane
CCLT@FT: Calcium/cobalt-based nanomodulator (Ca, Co)CO3-LND-TCPP@F127-TA
RmPLH: RBCm@Pt-CoNi layered double hydroxide
PATCM: Biomimetic nanoparticles loaded with semiconducting polymers, Atovaquone, and TMP195
G5-CHC-R: Nanovaccines conjugated with sonosensitizer, chenghai chlorin and resiquimod
GdNPs/Ce6-Ric: Tumor microenvironment-responsive nanoparticles encapsulating chlorin e6and Ricolinostat
DOXSFNPs: Chitosan hydrogel containing silk fibroin nanoparticles loaded with doxorubicin
Chl-MOF: *Chlorella* *vulgaris* (Chl) modified with metal‒organic framework nanoparticles
HM: Hybrid membrane
siRNA: Small interfering RNA
NBs: Nanobubbles
SPNH: Semiconducting polymer nanoparticles
EV: Extracellular vesicle
